# O/C Ratio‐Driven Fluorescence Enhancement in Cellulose‐Derived Carbon Quantum Dots: Mechanistic Insights into Reaction Pathways

**DOI:** 10.1002/advs.202510577

**Published:** 2025-10-06

**Authors:** Yarong Shi, Siyu Zhao, Xiheng Kang, Xinrui Chen, Xue Ou, Meng Nan Chong, Xueping Song, Zhanying Zhang

**Affiliations:** ^1^ Guangxi Key Laboratory of Clean Pulp & Papermaking and Pollution Control School of Light Industry and Food Engineering Guangxi University Nanning 530004 P. R. China; ^2^ Department of Chemical Engineering Monash University Malaysia Jalan Lagoon Selatan DE Bandar Sunway Selangor 47500 Malaysia; ^3^ Centre for Net‐Zero Technology Monash University Malaysia Jalan Lagoon Selatan DE Bandar Sunway Selangor 47500 Malaysia; ^4^ School of Mechanical Medical and Process Engineering Centre for Agriculture and the Bioeconomy Queensland University of Technology Brisbane Queensland 4000 Australia

**Keywords:** cellulose‐derived CQDs, Fe^3+^ detection, fluorescence enhancement, O/C ratio, synthesis mechanism

## Abstract

By regulating cellulose molecular weight through enzymatic hydrolysis to obtain different carbon precursors, three types of carbon quantum dots (CQDs) are synthesized via hydrothermal methods: cellulose enzyme‐hydrolyzed solids‐based carbon quantum dots (CES‐CQDs), cellulose enzyme‐hydrolyzed mixtures‐based carbon quantum dots (CEM‐CQDs), and cellulose hydrothermal degradation products‐based carbon quantum dots (CHD‐CQDs). By controlling the molecular weight of the cellulose precursor, the O/C ratio of the CQDs is systematically modulated from 0.25 to 0.61, resulting in a more than five‐fold increase in fluorescence intensity and an approximately seven‐fold improvement in quantum yield (QY). Density functional theory (DFT) calculations indicate that high O/C ratio enhances oscillator strength, thereby boosting fluorescence. Through a combination of experimental and density functional theory analyses, the formation mechanisms of cellulose‐derived CQDs are revealed to involve: primarily the auto‐etherification of 5‐hydroxymethylfurfural (5‐HMF), concurrently accompanied by esterification reactions between 5‐HMF and formic acid (FA), as well as aldol condensation reactions between 5‐HMF and levulinic acid (LA). This study elucidates the fundamental relationship between the molecular structure of cellulose precursors and the fluorescence properties of CQDs, providing a universal strategy for rationally designing high‐performance luminescent nanomaterials from renewable biomass.

## Graphical abstract

Three types of CQDs are prepared from cellulose and its derivatives via hydrothermal carbonization. An increase in the atomic O/C ratio from 0.25 to 0.61 results in a nearly seven‐fold enhancement in QY. DFT calculations verify this mechanism. Further, this study elucidates the formation pathways of cellulose‐derived CQDs and proposes a fluorescence optimization strategy through integrated experimental and DFT analyses.

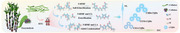

## 1 Introduction

Cellulose with a carbon content of 44%, is one of the three major biopolymers in lignocellulose and is also the most abundant biopolymer on earth.^[^ ^1^, ^2^ ^]^ Cellulose is a type of glucan and contains up to 49% oxygen that primarily exists in hydroxyl groups.^[^ ^3^ ^]^ At severe conditions such as high temperature and pressure, hydroxyl groups in cellulose can be dehydrated and the resulting intermediates are condensed, leading to the formation of various carbon structures.^[^ ^4^ ^]^ The hydroxyl groups in cellulose can also be converted into a variety of functional groups, such as aldehyde and ketones.^[^ ^3^, ^5^ ^]^ These features make cellulose being an attractive precursor for the synthesis of carbon quantum dots (CQDs), which also have many potential applications based on their fluorescence properties.^[^ ^6^, ^7^ ^]^

The fluorescence properties of CQDs are predominantly determined by two critical factors: the carbon core state and the surface state. In terms of the surface state, due to elements from VA and VIA (S, P, N, O) possessing lone electron pairs that significantly impact molecular dipole moments, bond lengths, spatial configurations, and electronic effects, they can signally enhance the fluorescence properties of CQDs.^[^ ^8^ ^]^ Elements such as S, P, and N are typically incorporated into lignocellulose‐based CQDs through doping,^[^ ^9^, ^10^, ^11^ ^]^ and their relatively low concentrations can substantially alter the physicochemical properties and optical performance of CQDs. However, oxygen and carbon elements form the fundamental structural framework of CQDs, typically accounting for 80% or more of their composition, and it remains unknown whether the ratio of O to C, affect the fluorescence properties of CQDs. Therefore, adjusting the O/C ratio of CQDs may serve as a viable approach to controlling the fluorescent properties of CQDs.

On the other hand, the performance of CQDs is usually inherited from the carbon source.^[^ ^12^, ^13^ ^]^ That is to say, adjusting the O/C ratio of the carbon source may affect the O/C ratio of CQDs, thus improving the performance of CQDs. Actually, for the catalytic or enzymatic degradation of biobased polymers, the change in molecular weight also accompanied by the change in oxygen content.^[^ ^14^, ^15^ ^]^ Moreover, controlling the molecular weight of the carbon source can significantly affect the performance of CQDs. For instance, our group has successfully improved the fluorescence properties of lignin‐based CQDs (L‐CQDs) only by controlling the lignin molecular weight with catalytic cleavage or solvent‐based fractionation approach.^[^ ^16^, ^17^ ^]^ However, how the molecular weight of the precursor affects the O/C ratio of CQDs and consequently influences their fluorescence performance remains unclear. To address this issue, we will design this study to investigate whether regulating the molecular weight of cellulose precursor through enzymatic hydrolysis can serve as an effective strategy to control the O/C ratio of the resulting CQDs, thus enhancing their fluorescence properties. We also aim to explore the formation mechanism of cellulose‐derived CQDs, thereby establishing a green and efficient pathway for controlling enzyme hydrolysis of cellulose and obtaining superior carbon source to manufacture high‐performance cellulose‐derived CQDs.

Unlike previous studies that improved the fluorescence properties of CQDs through heteroatom doping, this research achieves high‐performance cellulose‐based CQDs only by controlling the molecular weight of cellulose precursor with enzymatic hydrolysis method. Three types of CQDs will been synthesized: CES‐CQDs from solid residues after enzymatic hydrolysis of cellulose, CEM‐CQDs from the solid and liquid mixtures obtained by enzymatic hydrolysis of cellulose, and CHD‐CQDs from key hydrothermal intermediates of cellulose (5‐hydroxymethylfurfural (5‐HMF), furfural (F), formic acid (FA), and levulinic acid (LA)). Then, by systematically correlating the degree of polymerization (DP) of cellulose with the properties of the resulting CQDs, the relationship between the reduction of precursor molecular weight and the O/C ratio of the resulting CQDs, as well as the relationship between the O/C ratio and the fluorescence performance of CQDs will be established. Combined with DFT theoretical simulations, the mechanism by which the O/C ratio affects the fluorescence properties of CQDs will been further elucidated. Furthermore, the effects of cellulose hydrolysis products (FA, LA, and F on 5‐HMF) consumption during CQDs formation on the properties of CQDs will been investigated, proposing the formation mechanism of cellulose‐derived CQDs with high fluorescence, which will put forward an efficient strategy for controlling enzyme hydrolysis and obtaining superior carbon source to manufacture high‐performance cellulose‐derived CQDs. Additionally, the potential of these CQDs as fluorescent sensors for metal ion detection will also be demonstrated. This study not only provides a novel strategy for preparing high‐performance CQDs by controlling precursor but also offers fundamental insights into the significance of O/C ratio of CQDs in influencing the luminescent properties of cellulose‐derived CQDs.

## 2 Results and Discussion

### 2.1 Analysis of Enzymatic Cellulose Characteristics

**Figure 1:**
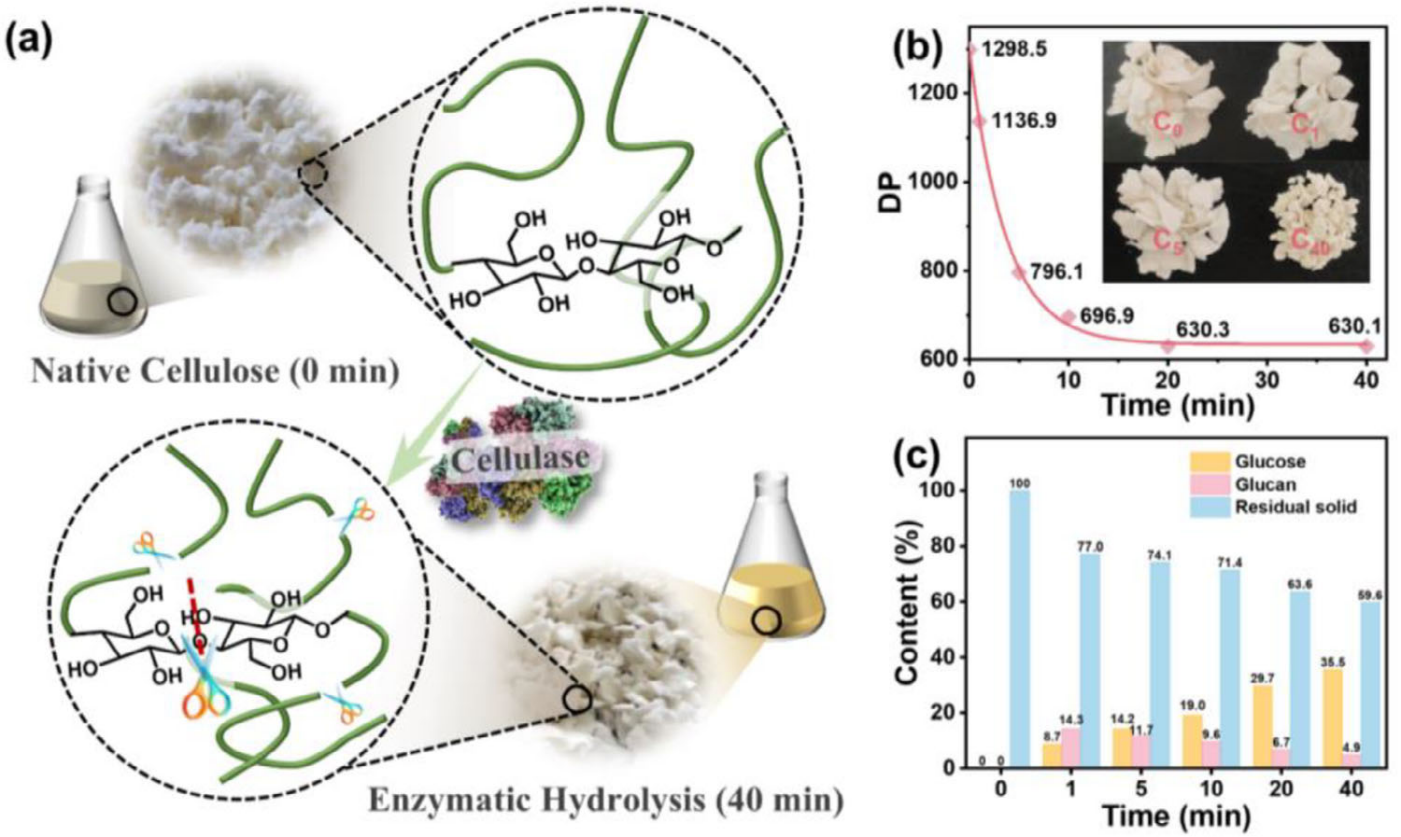
Analysis of cellulose enzymatic hydrolysis products. a) The changes during the enzymatic hydrolysis of cellulose. b) DP of residual cellulose (The inset is a photo of residual air‐dried pulp after different enzymatic hydrolysis times). c) Content of glucose, polysaccharides, and residual cellulose in the mixture after enzymatic hydrolysis.

In the experimental part, the enzymatic hydrolysis of cellulose and the synthesis of three kinds of CQDs are introduced in detail. **Figure** **1**a shows the changes during the enzymatic hydrolysis of cellulose. As shown in Figure 1b, during the initial 5 min of enzymatic treatment of pulp cellulose, the reaction is at its most intense, causing a sharp decline in the DP of cellulose from 1298.5 to 796.1. After 5 min, the enzyme hydrolysis rate gradually decreases. When the enzyme hydrolysis time extends from 10 and 40 min, the DP of cellulose from 696.9 to 630.1, representing a modest reduction of only 5.14%. It is well known that the molecular weight of cellulose is positively correlated with its DP. For consistency in expression, subsequent analyses will uniformly use DP as the metric. As can be seen from Figure 1c, during the enzymatic hydrolysis process, the glucose content in the system increases from 0% to 35.5%, while the polysaccharide content decreases from 14.3% at 1 min to 4.9% at 40 min. This indicates that enzymatic hydrolysis cleaves some 1,4‐β‐glycosidic bonds in the cellulose structure, leading to the release of low molecular weight polysaccharides and glucose. At the same time, under the action of the enzyme, the polysaccharide is further degraded into glucose. Therefore, after enzymatic hydrolysis, undegraded cellulose, polysaccharides, and glucose coexist in the system. For the chemical structure changes and elemental composition changes of cellulose with different degrees of enzymatic hydrolysis, please refer to Figures S1 and S2 and Equation S1 (Supporting information).

### 2.2 Property Analysis of CES‐CQDs and CEM‐CQDs

#### 2.2.1 Fluorescence Characteristics Analysis of CES‐CQDs and CEM‐CQDs

**Figure 2:**
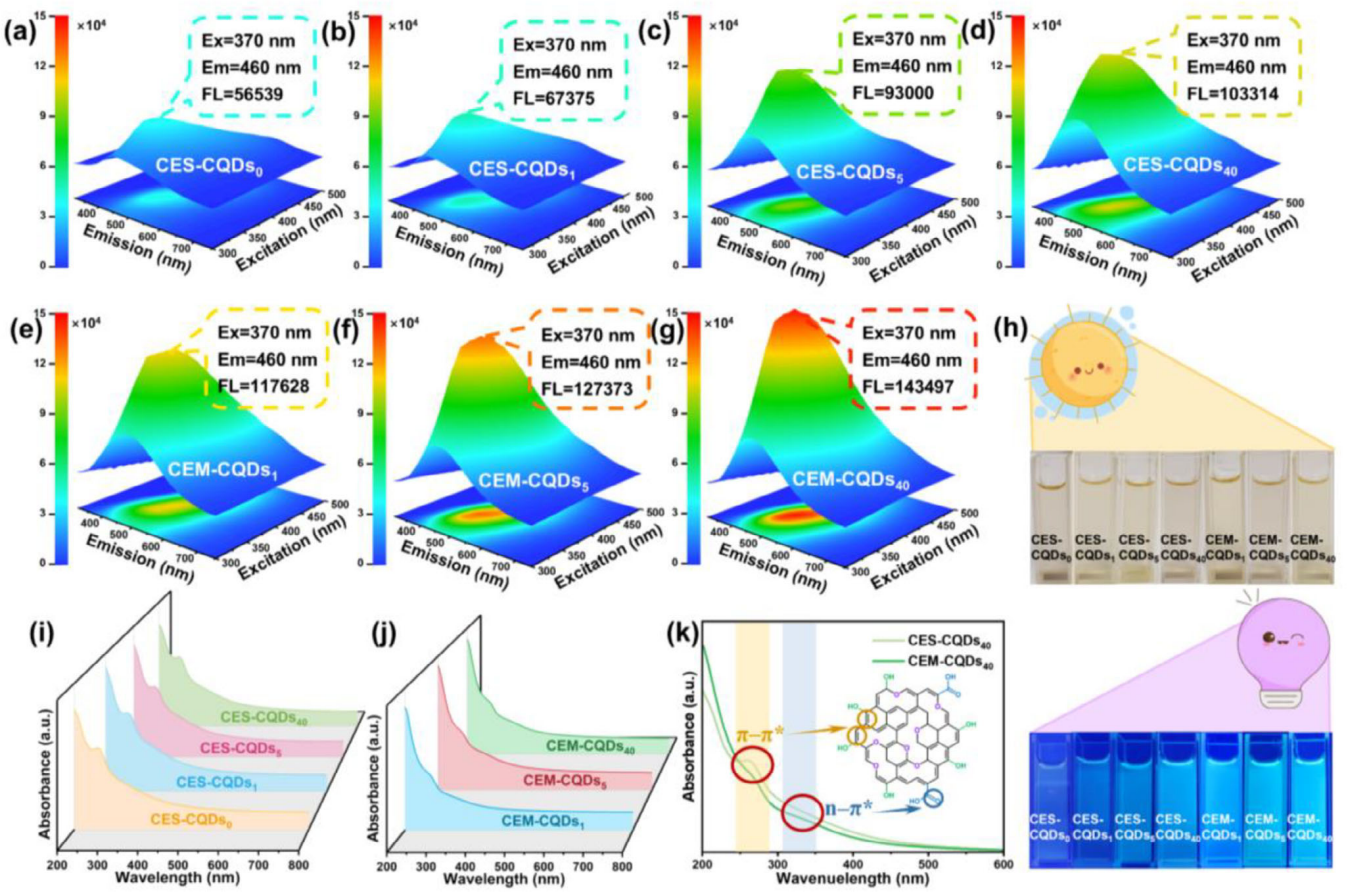
Optical properties of CQDs. 3D fluorescence spectra: a) CES‐CQDs_0_, b) CES‐CQDs_1_, c) CES‐CQDs_5_, d) CES‐CQDs_40_, e) CEM‐CQDs_1_, f) CEM‐CQDs_5_, g) CEM‐CQDs_40_. h) The pictures of CQDs solution under natural light and 365nm UV light. UV–vis: i) CES‐CQDs, j) CEM‐CQDs, and k) Schematic diagram of absorption band positions.

The optical properties of CQDs were studied using fluorescence spectroscopy, QY, and UV–vis spectroscopy. **Figure** **2**a–g display the 3D fluorescence spectra of the CES‐CQDs and CEM‐CQDs at a concentration of 0.1 mg mL^−1^. All CQDs exhibit an optimal excitation wavelength of 370 nm, with a corresponding optimal emission wavelength of 460 nm. It can be seen from Table S1 (Supporting Information) that with the extension of enzymatic hydrolysis time, both the QY and fluorescence intensity of CES‐CQDs and CEM‐CQDs are increased. Under the same enzymatic hydrolysis time, the fluorescence performance of CEM‐CQDs is superior to that of CES‐CQDs. Compared with CES‐CQDs_0_, CEM‐CQDs_40_ exhibits less than a three‐fold enhancement in fluorescence intensity and an approximately four‐fold increase in QY. This may be attributed to the improved fluorescence performance of CEM‐CQDs due to the richer surface hydroxyl groups and enhanced p‐π conjugation compared with CES‐CQDs.^[^ ^18^ ^]^ At the same time, each CQDs has only one emission center, indicating that the difference in DP of cellulose and the presence of glucose and low molecular weight polysaccharides in the enzymatic hydrolysate do not affect the optimal excitation wavelength and emission center of CQDs. In addition, from the 2D fluorescence spectrum in Figure S3 (Supporting Information), it is found that the seven CQDs all exhibit the “excitation wavelength dependence” characteristic of conventional CQDs.^[^ ^17^ ^]^ As the excitation wavelength increases, the emission wavelength of both CES‐CQDs and CEM‐CQDs shows a slight red shift. This is mainly due to the relaxatin of the surface polar groups (hydroxyl and carboxyl) of CQDs in polar solvents such as deionized water, resulting in a “giant red edge effect.”^[^ ^19^ ^]^ Figure 2h shows the corresponding CQDs solution under natural light and 365 nm ultraviolet light. Under natural light, the CQDs aqueous solution appears light yellow, and the solution emits bright blue fluorescence under 365 nm ultraviolet light. The fluorescence brightness tends to increase, with CEM‐CQDs_40_ being the brightest. As shown in Figure 2h–j, the UV–vis spectra indicate that both CES‐CQDs and CEM‐CQDs exhibit absorption in the range of 200–600 nm. The peak observed below 300 nm in the UV–vis spectra corresponds to the π–π* transition of the aromatic ring in the CQDs skeleton. A weaker absorption peak at 330 nm is attributed to the n‐π* transition of the aldehyde and ketone structures,^[^ ^20^ ^]^ suggesting the presence of C═O groups on the surface of CQDs. The absorption peak positions of all CQDs remain consistent, suggesting that the types of intramolecular electronic energy level transitions induced by CQDs under UV–vis light irradiation are identical.

#### 2.2.2 Chemical Properties Analysis of CES‐CQDs and CEM‐CQDs

**Figure 3:**
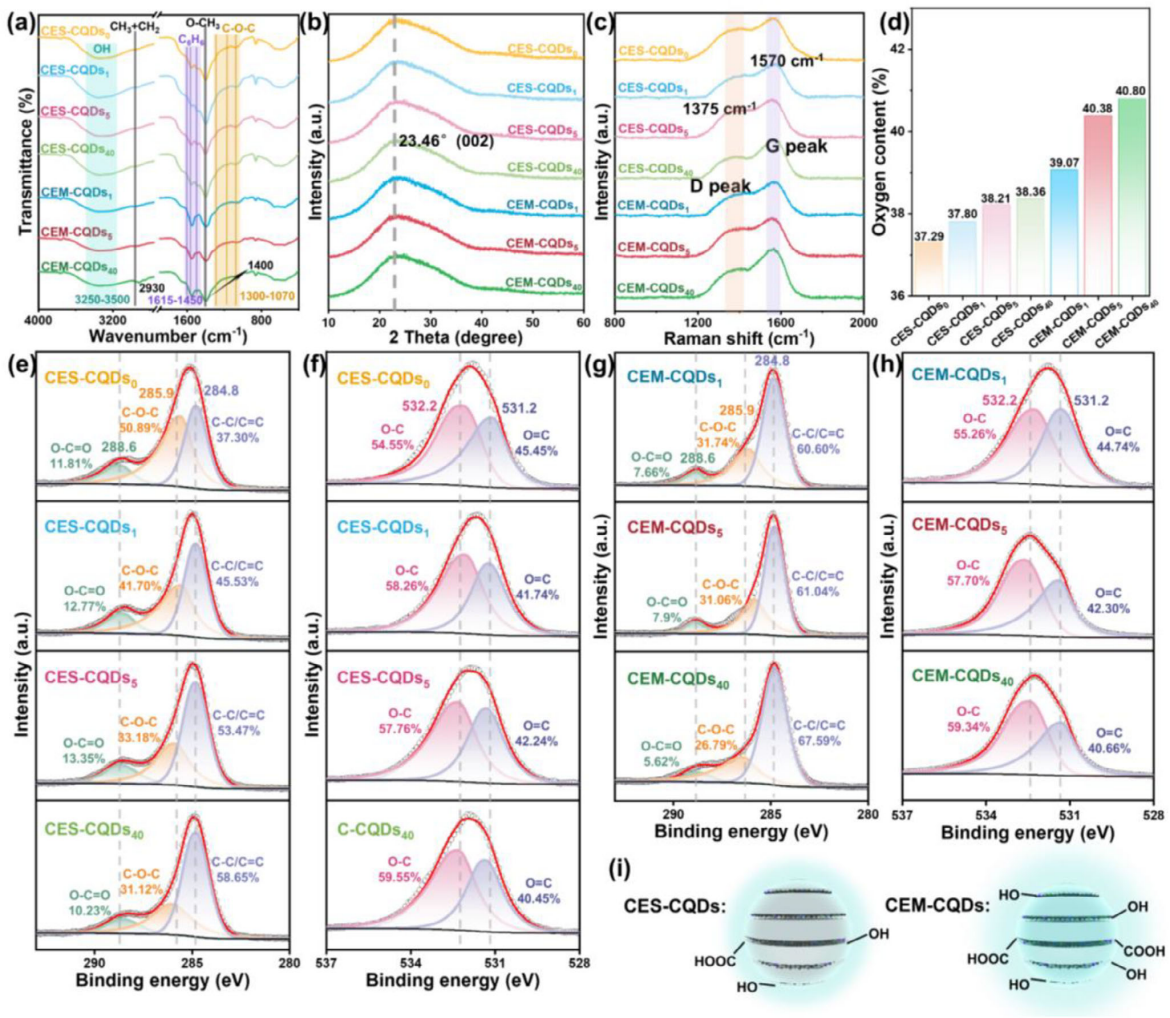
Chemical properties of CQDs. a) FTIR spectra; b) XRD spectra; c) Raman spectra; d) EA(O). e) XPS‐C1s high‐resolution spectra: e) CES‐CQDs and f) CEM‐CQDs; XPS‐O 1*s* high‐resolution spectra: g) CES‐CQDs and h) CEM‐CQDs. i) Schematic diagram of CQDs structure.

As shown in **Figure** **3**a, the FTIR spectra of CES‐CQDs and CEM‐CQDs exhibit similar peak positions, which include the following functional groups: ─OH groups (3250–3500 cm^−1^), ─CH_3_ and ─CH_2_─ (2930 cm^−1^), C═O (1697 cm^−1^), C═C (1660 cm^−1^), benzene ring (1615–1450 cm^−1^), O─CH_3_ (1400 cm^−1^), and C─O─C (1300–1050 cm^−1^).^[^ ^21^ ^]^ The XRD results are shown in Figure 3b. All CQDs exhibit a broad diffraction peak at 2θ = 24°, which corresponds to the (002) plane of graphite with an interlayer spacing of 0.34 nm, indicating the presence of graphitic structure.^[^ ^22^ ^]^ The interlayer spacing of CQDs is close to that of graphite (≈0.35 nm), suggesting that CQDs possess a graphite‐like core structure and excellent crystallinity. The Raman spectra results are shown in Figure 3c. CQDs exhibit two characteristic peaks: the graphite G band at ≈1570 cm^−1^ and the disordered D band at ≈1375 cm^−1^.^[^ ^23^ ^]^ The intensity ratios (I_D_/I_G_) of CES‐CQDs_0_, CES‐CQDs_1_, CES‐CQDs_5_, and CES‐CQDs_40_ are 0.73, 0.70, 0.66, and 0.62, respectively; the I_D_/I_G_ of CEM‐CQDs_1_, CEM‐CQDs_5_, and CEM‐CQDs_40_ are 0.72, 0.63, and 0.58, respectively. These results indicate that as the decrease of cellulose DP, the graphitization degree of the prepared CQDs gradually increases. Furthermore, under the same degree of enzymatic hydrolysis, the graphitization degree of CEM‐CQDs is consistently higher than that of CES‐CQDs. This is because glucose and small molecular weight polysaccharides in the reactants degrade faster than structurally stable cellulose under thermal conditions. Under identical hydrothermal conditions, the depolymerization process requires less time while the polymerization process takes longer, facilitating the rearrangement of carbon atoms.

EA analysis in Figure 3d indicates that the oxygen content in CQDs increases with enzymatic hydrolysis time. Moreover, at the same enzymatic hydrolysis duration, CEM‐CQDs exhibit higher oxygen content than CES‐CQDs. XPS was further used to study the surface functional group composition and valence state of the elements contained in CES‐CQDs and CEM‐CQDs. The O/C ratios of CES‐CQDs_0_, CES‐CQDs_1_, CES‐CQDs_5_, and CES‐CQDs_40_ are 0.25, 0.27, 0.28, and 0.38, respectively; the O/C ratios of CEM‐CQDs_1_, CEM‐CQDs_5_, and CEM‐CQDs_40_ are 0.38, 0.47, and 0.52, respectively. By comparison, it is found that under the same enzymatic hydrolysis time, the content of O element in CEM‐CQDs is higher than that in CES‐CQDs. As the DP of the carbon precursor cellulose decreases, the proportion of O element in both CEM‐CQDs and CES‐CQDs increases. The increase in O element content is attributed to the enzymatic hydrolysis of cellulose, which cleaves the 1,4‐β‐glycosidic bonds and reduces the DP. Simultaneously, the hydrothermal precursor exposes more O elements, leading to an increase in oxygen‐containing functional groups on the surface of CQDs. In addition, the high‐resolution energy spectra of C 1*s* and O 1*s* in CES‐CQDs were scanned, and the results are shown in Figure 3e,f. The C 1*s* high‐resolution energy spectra of the four CES‐CQDs show three distinct peaks. The peak at 284.8 eV is attributed to C─C/C═C, the peak at 285.90 eV is attributed to C─O─C, and the peak at 288.6 eV is attributed to O─C═O in carboxyl and ester groups.^[^ ^24^ ^]^ The O 1*s* high‐resolution energy spectra of the four CES‐CQDs show two distinct peaks. The peak at 531.2 eV is attributed to O═C in carboxyl and ester groups, while the peak at 532.2 eV is attributed to O─C in phenolic hydroxyl and ether bonds.^[^ ^25^ ^]^ From the C 1*s* high‐resolution spectra, it can be seen that the content of C─C/C═C in CES‐CQDs_0_, CES‐CQDs_1_, CES‐CQDs_5_, and CES‐CQDs_40_ increases from 37.30% to 58.65%. From the O 1*s* high‐resolution spectra, it is observed that the ratio of O─C to O═C remains relatively stable. Additionally, the O─C content in all four CES‐CQDs is higher than that of O═C. These results show that as the DP of cellulose decreases, the degree of graphitization of the CES‐CQDs increases. At the same time, the exposure of more O elements leads to the formation of more electron‐donating groups, represented by hydroxyl groups, on the surface of the CES‐CQDs during the hydrothermal reaction. Figure 3g,h shows the C 1*s* and O 1*s* high‐resolution spectra of CEM‐CQDs. Compared with CES‐CQDs, the C 1*s* high‐resolution spectra of CEM‐CQDs show that, with the extension of enzymatic hydrolysis time, the structural change trends of CEM‐CQDs and CES‐CQDs are essentially the same. The ratio of C─C/C═C increases from 60.60% to 67.59%, indicating that, with the extension of enzymatic hydrolysis time, the graphitization degree of CEM‐CQDs also increases. At the same enzymatic hydrolysis time, the C─C/C═C ratio of CEM‐CQDs is higher than that of the corresponding CES‐CQDs, indicating that the degree of graphitization of CEM‐CQDs is higher than that of CES‐CQDs, which is consistent with the conclusion obtained from Raman analysis. In the O 1*s* high‐resolution spectra of CEM‐CQDs, the O‐C content shows an upward trend with the extension of enzyme hydrolysis time. The O─C content of the three CEM‐CQDs is higher than the O═C content, indicating the presence of oxygen‐containing functional groups, primarily hydroxyl groups, on the surface of CEM‐CQDs, which is consistent with CES‐CQDs. Furthermore, the distribution of functional groups on the surfaces of CES‐CQDs and CEM‐CQDs is shown in Figure 3i. The surface structural groups of CEM‐CQDs are consistent with those of CES‐CQDs, inheriting the high ─OH content characteristic of cellulose. However, the ─OH content in CEM‐CQDs is higher than that in CES‐CQDs.

#### 2.2.3 Physical Property Analysis of CES‐CQDs and CEM‐CQDs

**Figure 4:**
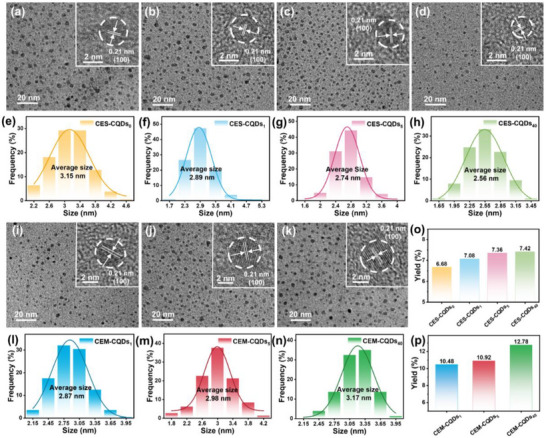
Physical properties of CQDs. TEM images: a) CES‐CQDs_0_, b) CES‐CQDs_1_, c) CES‐CQDs_5_, d) CES‐CQDs_40_, i) CEM‐CQDs_1_, j) CEM‐CQDs_5_, and k) CEM‐CQDs_40_. Particle size distribution: e) CES‐CQDs_0_, f) CES‐CQDs_1_, g) CES‐CQDs_5_, h) CES‐CQDs_40_, l) CEM‐CQDs_1_, m) CEM‐CQDs_5_, and n) CEM‐CQDs_40_. Yield: o) CES‐CQDs and p) CEM‐CQDs.

The microstructure and particle size distribution of CQDs were studied by TEM. From **Figure** **4**a–d,i–k, it is evident that the CQDs are spherical nanoparticles with uniform distribution, demonstrating their resistance to agglomeration in water and excellent water dispersibility. At higher resolution (inset), all CQDs exhibit distinct lattice fringes with a spacing of 0.21 nm, corresponding to the graphite (100) crystal plane, which confirms their graphite‐like structure.^[^ ^26^ ^]^ The diameters of more than 100 CQDs particles were randomly measured using Nano Measurer software to determine their average particle size distribution, as shown in Figure 4e–h,l–n. The average particle sizes of CES‐CQDs_0_, CES‐CQDs_1_, CES‐CQDs_5_, and CES‐CQDs_40_ are 3.15, 2.89, 2.74, and 2.56 nm, respectively, indicating that, with the decrease in the DP of cellulose, the average particle size of CES‐CQDs shows a slight decreasing trend. The average particle sizes of CEM‐CQDs_1_, CEM‐CQDs_5_, and CEM‐CQDs_40_ are 2.87, 2.98, and 3.17 nm, respectively. Indicating that, with the decrease in the DP of cellulose, the average particle size of CEM‐CQDs tends to increase, which is opposite to the trend observed for CES‐CQDs. This difference occurs because enzymatic hydrolysis generates abundant small‐molecule precursors (glucose, polysaccharide) that enhance aggregation in CEM‐CQDs preparation progress, while direct pyrolysis of low‐polymerization cellulose yields limited and unstable fragments that fail to coalesce effectively to form CES‐CQDs. From the statistics of CQDs production in Figure 4o,p, with the extension of enzymatic hydrolysis time, the yields of prepared CES‐CQDs and CEM‐CQDs both show an upward trend. At the same enzymatic hydrolysis time, the yield of CEM‐CQDs is higher than that of CES‐CQDs. Taking CES‐CQDs_40_ and CEM‐CQDs_40_, with the highest yield, as examples, the yield of CES‐CQDs_40_ is 7.42%, and the yield of CEM‐CQDs_40_ is 12.78%, which is 1.72 times that of the former.

#### 2.2.4 Relationship Between Oxygen‐Containing Functional Groups and Fluorescence Properties of CQDs

**Figure 5:**
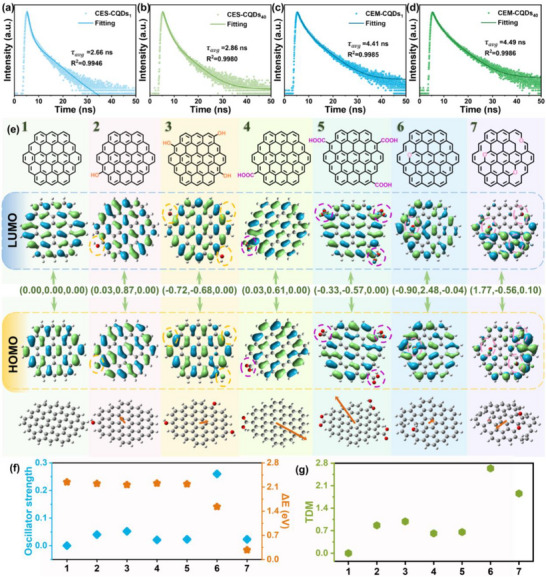
Fluorescence lifetime: a) CES‐CQDs_1_, b) CES‐CQDs_40_, c) CEM‐CQDs_1_ and, d) CEM‐CQDs_40_. Computational analysis of a simple molecular model of the CQDs layer structure: e) Molecular model structure and calculated frontier orbitals and transition dipole moments, where the direction and magnitude of the dipole moment are indicated by orange arrows; f) Oscillator energy (blue) and band gap ΔE (orange) of the molecular model; g) Magnitude of the transition dipole moment of the molecular model.

As demonstrated by the above analysis, among all CES‐CQDs and CEM‐CQDs prepared from enzymatically hydrolyzed cellulose, the samples prepared after 40 min of cellulolytic hydrolysis (CES‐CQDs_40_ and CEM‐CQDs_40_) exhibit the highest fluorescence intensity and QY within their respective categories. In contrast, the shortest cellulolytic hydrolysis time (1 min) results in the lowest fluorescence intensity and QY for CES‐CQDs_1_ and CEM‐CQDs_1_, making them the least efficient among all cellulolytic enzymolysis‐derived samples. To further elucidate the fluorescence mechanism of CQDs, the luminescence efficiency is investigated in relation to their chemical properties. At an excitation wavelength of 365 nm and an optimal emission wavelength of 460 nm, the time‐resolved photoluminescence spectra of four representative samples, CES‐CQDs_1_, CES‐CQDs_40_, CEM‐CQDs_1_, and CEM‐CQDs_40_, were tested, and the fluorescence lifetimes of the four CQDs were analyzed. The results are shown in **Figure** **5**a–d, and the relevant data are shown in Table S2 (Supporting Information) and specific analysis in supporting materials (Equations S2 and S3, Supporting Information). The average lifetimes of CES‐CQDs_1_, CES‐CQDs_40_, CEM‐CQDs_1_, and CEM‐CQDs_40_ are 2.66, 2.86, 4.41, and 4.49 ns, respectively. CES‐CQDs typically exhibit short‐lived fluorescence decay, and their fluorescence primarily originates from the carbon core state; whereas CEM‐CQDs generally demonstrate long‐lived fluorescence decay, and their fluorescence is mainly attributed to the surface state (high hydroxyl content). The above results demonstrate that compared with CES‐CQDs, the O/C ratio and oxygen‐containing functional groups on the surface of CEM‐CQDs are increased (Figure 2), and the fluorescence intensity of CEM‐CQDs are significantly higher than those of CES‐CQDs (Figure 3). This suggests that the oxygen‐containing functional groups on the surface of CEM‐CQDs have a greater influence on their fluorescence properties.

In order to further clarify the influence of different oxygen‐containing functional groups on the surface of CQDs on their fluorescence properties, seven CQDs models with basically the same molecular weight are constructed: molecule 1 has no oxygen‐containing functional groups on the edge, molecule 2 has one phenolic hydroxyl group on the edge, molecule 3 has three phenolic hydroxyl groups on the edge, molecule 4 has one carboxyl group on the edge, molecule 5 has three carboxyl groups on the edge, molecule 6 has one ether bond, and molecule 7 has three ether bond. It should be noted that these are simplified models designed to expound the effect of individual functional groups; actual CQDs possess a complex distribution of these and other oxygenated groups species. DFT calculations are performed to compare the calculated results of oscillator energy and transition dipole moment (TDM) of each molecular model. In Figure 5e, each column represents a molecular model, which includes the structural formula of the model, the LUMO/HOMO electron cloud distribution diagram, and the magnitude and direction of the transition dipole moment from top to bottom. The oscillator energies of molecules 2, 3, 4, 5, 6 and 7 in Figure 5f are 0.040, 0.052, 0.021, 0.023, 0.26 and 0.023, respectively; the TDM values of molecules 2, 3, 4, 5, 6 and 7 in Figure 5g are 0.866, 0.987, 0.616, 0.657, 2.636 and 1.856, respectively, which are all higher than those of molecule 1 (both oscillator energy and TDM are 0). This indicates that the increase in the number of oxygen‐containing functional groups on the edge of CQDs molecules enhances their TDM and oscillator energy. A large TDM and high oscillator energy leads to an increase in the QY of the molecules,^[^ ^27^ ^]^ thereby making CQDs exhibit a good fluorescence performance. This is consistent with the experimental results.

During the hydrothermal synthesis of CQDs using cellulose and its derivatives as carbon source, the primary processes involved are carbonization (formation of the CQDs framework) and oxygen consumption (in the form of H_2_O, CO_2_, etc.).^[^ ^28^ ^]^ The resulting CQDs exhibit abundant oxygen‐containing functional groups, including C─OH, C─COOH, and C─O─C. C─OH and C─COOH are the primary contributors of oxygen atoms. They directly introduce oxygen atoms to the surface of CQDs. During the later stages of hydrothermal carbonization or surface oxidation, residual or newly formed hydroxyl and carboxyl groups also increase the O/C ratio of CQDs. The presence of C─OH helps to reduce the non‐radiative transition process of CQDs, thereby increasing QY.^[^ ^19^, ^29^, ^30^ ^]^ Additionally, C─COOH can act as electron acceptors or donors to form charge transfer complexes with carbon atoms in CQDs, further enhancing fluorescence emission.^[^ ^31^, ^32^ ^]^ C─O─C primarily originates from the residual breakdown of original cellulose glycosidic bonds. During HTC, the cleavage of cellulose β‐1,4‐glycosidic bonds often serves as a key step in deoxygenation reactions.^[^ ^33^ ^]^ These reactions lead to the release of oxygen‐containing small molecules, thereby consuming oxygen atoms in the system. Therefore, the presence of C─O─C groups is often accompanied by a consumption of oxygen atoms, and an increase in their proportion typically reduces the O/C ratio of CQDs. A small number of C─O─C bonds may lead to more thorough carbonization of the carbon core and increase the sp^2^ domain, but too many C─O─C bonds will cause some defects in the carbon skeleton of CQDs, which is not conducive to fluorescence emission.^[^ ^34^ ^]^

In general, the carbonization and oxygen consumption that occur during the hydrothermal preparation of CQDs from cellulose are accompanied by the generation and transformation of surface functional groups. Among these, as the molecular weight of the precursor decreases, the content of C─OH and C─COOH groups which are key factors in enhancing the O/C ratio increases, and the O/C ratio exhibits a positive correlation with the fluorescence properties of CQDs. Therefore, the enhanced fluorescence phenomena of CEM‐CQDs are primarily attributed to their high O/C ratio.

### 2.3 Relationship Between the Change of Intermediate Products from Cellulose Hydrothermal Carbonization and Properties of CHD‐CQDs

Based on the study of the fluorescence properties and physical‐chemical characteristics of CES‐CQDs and CEM‐CQDs, it can be clearly demonstrated that the O/C value has a significant positive correlation with the fluorescence properties of cellulose‐derived CQDs. However, the study of CES‐CQDs and CEM‐CQDs alone has certain limitations: it is impossible to clearly identify the effect of specific intermediate products (5‐HMF, F, FA, and LA) on the fluorescence properties of CQDs during the hydrothermal process of cellulose.^[^ ^35^ ^]^ Therefore, it is necessary to further study the effect of these specific intermediate products of cellulose on improving the O/C ratio of CQDs, thereby enhancing their fluorescence performance.

**Figure 6:**
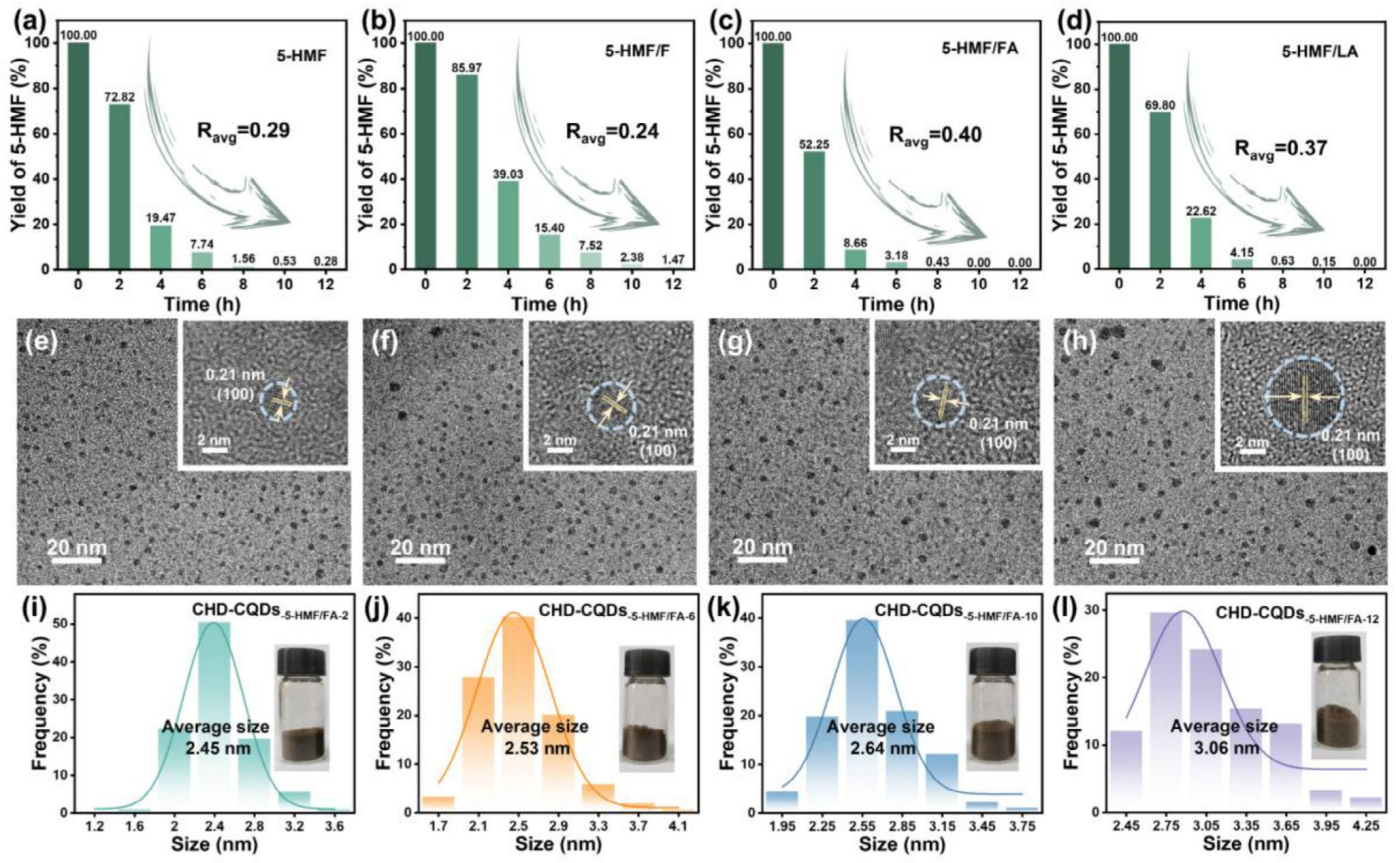
Consumption rates of the main hydrolysis products of cellulose: a) 5‐HMF, b) 5‐HMF/F, c) 5‐HMF/FA, and d) 5‐HMF/LA. TEM images of CHD‐CQDs_‐5‐HMF/FA_: e) CHD‐CQDs_‐5‐HMF/FA‐2_, f) CHD‐CQDs_‐5‐HMF/FA‐6_, g) CHD‐CQDs_‐5‐HMF/FA‐10_, and h) CHD‐CQDs_‐5‐HMF/FA‐12_. Particle size distribution of CHD‐CQDs3 (the inner figures are the actual picture of the corresponding CQDs): i) CHD‐CQDs_‐5‐HMF/FA‐2_, j) CHD‐CQDs_‐5‐HMF/FA‐6_, k) CHD‐CQDs_‐5‐HMF/FA‐10_, and l) CHD‐CQDs_‐5‐HMF/FA‐12_.

When CQDs are prepared using 5‐HMF, F, FA, and LA as carbon sources, it is found that spherical CQDs form when 5‐HMF is present in the hydrothermal system. When only F is used, CQDs also form, but the yield is extremely low, and they readily absorb moisture, resulting in a brown paste (as shown in Figure S4, Supporting Information). However, when FA and LA are used alone as precursors, CQDs do not form; this suggests that 5‐HMF is a key intermediate for the preparation of cellulose‐derived CQDs. In this study, CHD‐CQDs_‐5‐HMF_, CHD‐CQDs_‐5‐HMF/F_, CHD‐CQDs_‐5‐HMF/FA_, and CHD‐CQDs_‐5‐HMF/LA_ are prepared using 5‐HMF, 5‐HMF/F, 5‐HMF/FA and 5‐HMF/LA as carbon precursors, respectively. The relationship between the effects of mainly cellulose hydrothermal degradation products (F, FA, and LA) on the consumption of 5‐HMF with the physicochemical and fluorescence properties of CHD‐CQDs is investigated, and the results are shown in **Figure** **6**a–d.

Calculations through Equation S4 (Supporting Information) show that when the carbon precursor is only 5‐HMF, the average hourly consumption rate is 0.29. When the carbon precursor is 5‐HMF/F, the average hourly consumption rate is 0.24, which is lower than 0.29, indicating that the addition of F inhibits the conversion of 5‐HMF to CQDs or that 5‐HMF and F compete with each other. When the carbon precursor is 5‐HMF/FA, the average hourly consumption rate increases to 0.40, and when the carbon precursor is 5‐HMF/LA, it rises to 0.37, indicating that both FA and LA promote the consumption of 5‐HMF. When the hydrothermal time reaches 10 h, 5‐HMF in all samples is almost completely consumed.

Based on the fact that FA plays the most significant role in promoting the formation of CQDs during the 5‐HMF process, CHD‐CQDs_‐5‐HMF/FA_ were taken for example, its formation process is systematically investigated by TEM. As illustrated in Figure 6e–h, CHD‐CQDs_‐5‐HMF/FA_ maintain a uniform distribution of spherical nanoparticles throughout the hydrothermal process. Analysis of particle size distribution (Figure 6i–l) demonstrates a gradual increase in average particle diameter from 2.45 ± 0.15 nm (CHD‐CQDs_‐5‐HMF/FA‐2_) to 3.06 ± 0.18 nm (CHD‐CQDs_‐5‐HMF/FA‐12_) with extended hydrothermal duration. This indicates that CHD‐CQDs_‐5‐HMF/FA_ is formed by gradual polymerization when small molecules (5‐HMF and FA) are used as precursors for hydrothermal reaction. This observation is further supported by the consistent particle size evolution patterns observed in CHD‐CQDs_‐5‐HMF_ and CHD‐CQDs_‐5‐HMF/LA_, as presented in Figures S5 and S6 (Supporting Information).

**Figure 7:**
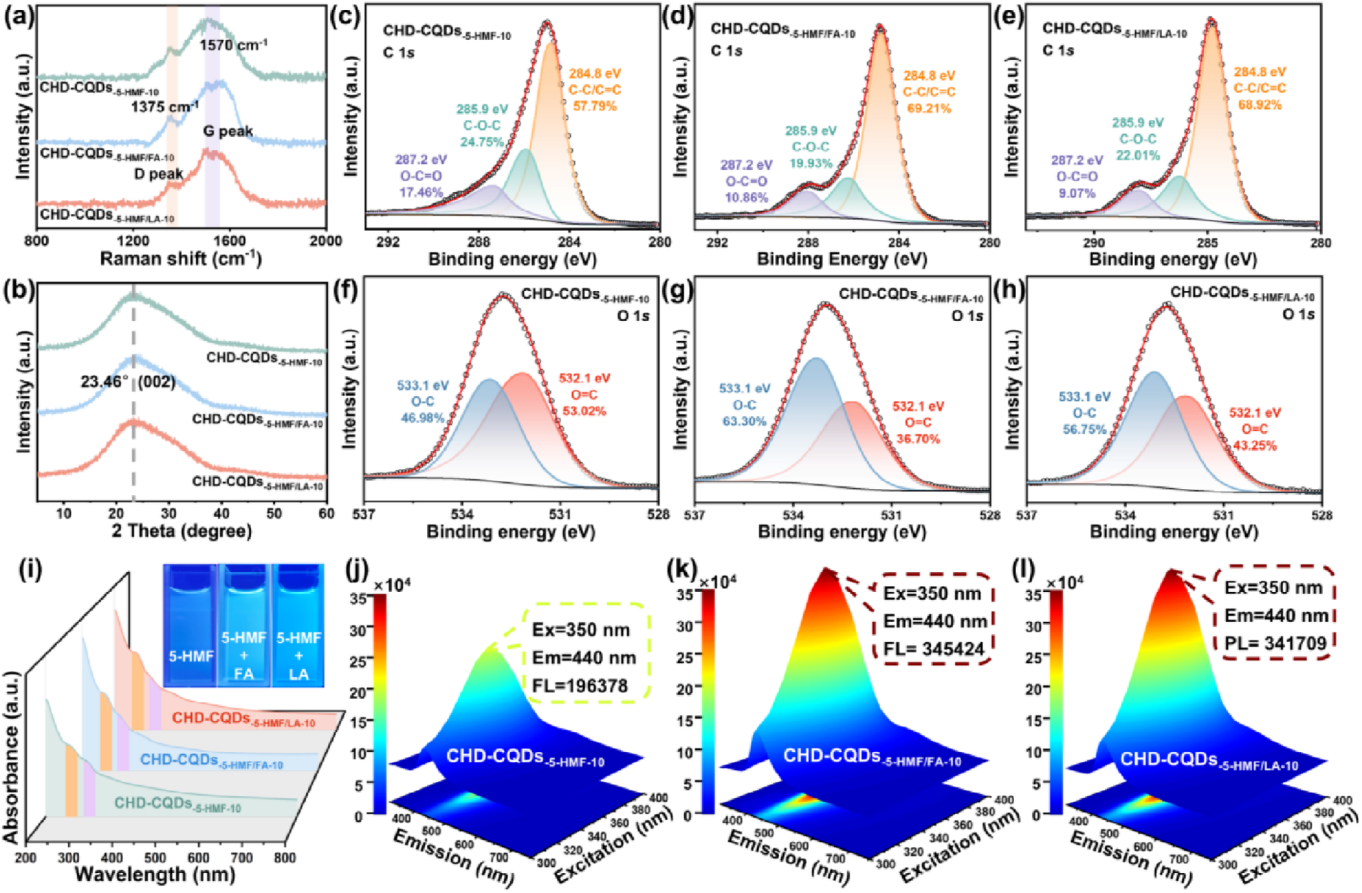
a) Raman spectra and b) XRD of CHD‐CQDs_‐5‐HMF‐10_, CHD‐CQDs_‐5‐HMF/FA‐10_, and CHD‐CQDs_‐5‐HMF/FA‐10_. C 1*s* high‐resolution XPS spectra: c) CHD‐CQDs_‐5‐HMF‐10_, d) CHD‐CQDs_‐5‐HMF/FA‐10_, and e) CHD‐CQDs_‐5‐HMF/FA‐10_; O 1*s* high‐resolution XPS spectra: f) CHD‐CQDs_‐5‐HMF‐10_, g) CHD‐CQDs_‐5‐HMF/FA‐10_, and h) CHD‐CQDs_‐5‐HMF/FA‐10_. i) UV–vis of CHD‐CQDs_‐5‐HMF‐10_, CHD‐CQDs_‐5‐HMF/FA‐10_, and CHD‐CQDs_‐5‐HMF/LA‐10_ (The inner figures correspond to their images in solution under UV illumination, respectively). 3D fluorescence spectra: j) CHD‐CQDs_‐5‐HMF‐10_, k) CHD‐CQDs_‐5‐HMF/FA‐10_, and l) CHD‐CQDs_‐5‐HMF/LA‐10_.

Represented by CHD‐CQDs_‐5‐HMF‐10_, CHD‐CQDs_‐5‐HMF/FA‐10_, and CHD‐CQDs_‐5‐HMF/LA‐10_, the physicochemical properties and fluorescence performance of CHD‐CQDs were investigated. **Figure** **7**a presents the Raman spectra of CHD‐CQDs_‐5‐HMF‐10_, CHD‐CQDs_‐5‐HMF/FA‐10_, and CHD‐CQDs_‐5‐HMF/LA‐10_. The corresponding intensity ratios of the D‐band to G‐band (I_D_/I_G_) are measured as 0.65, 0.52, and 0.57, respectively. Among these, CHD‐CQDs_‐5‐HMF/FA‐10_ exhibit the highest degree of graphitization. The Raman spectra for all CHD‐CQDs (include CHD‐CQDs_‐5‐HMF_, CHD‐CQDs_‐5‐HMF/F_, CHD‐CQDs_‐5‐HMF/FA_, and CHD‐CQDs_‐5‐HMF/LA_) are provided in Figure S7 (Supporting Information). As the hydrothermal duration increased, the graphitization degree of CHD‐CQDs demonstrated an overall upward trend. The XRD patterns of CHD‐CQDs are depicted in Figure 7b and Figure S8 (Supporting Information) (include CHD‐CQDs_‐5‐HMF_, CHD‐CQDs_‐5‐HMF/F_, CHD‐CQDs_‐5‐HMF/FA_, and CHD‐CQDs_‐5‐HMF/LA_). These patterns are consistent with the peak profiles observed for CES‐CQDs and CEM‐CQDs in Figure 2b, featuring a broad peak at 2θ = 24°. This indicates that CHD‐CQDs possess a graphite‐like core structure and exhibit good crystallinity.

The surface functional groups composition of CHD‐CQDs_‐5‐HMF‐10_, CHD‐CQDs_‐5‐HMF/FA‐10_, and CHD‐CQDs_‐5‐HMF/LA‐10_ are analyzed by XPS, and the O/C ratios are 0.44, 0.61, and 0.53, respectively, the O/C ratio of CEM‐CQDs_40_ is 0.52. These results indicate that, compared with CES‐CQDs and CEM‐CQDs, the O/C ratio in CHD‐CQDs is further elevated. High‐resolution XPS spectra of C 1*s* and O 1*s* for CHD‐CQDs_‐5‐HMF‐10_, CHD‐CQDs_‐5‐HMF/FA‐10_, and CHD‐CQDs_‐5‐HMF/LA‐10_ are presented in Figure 7c–h. The peak positions in the C 1*s* and O 1*s* spectra of these CHD‐CQDs align with those of CES‐CQDs and CEM‐CQDs shown in Figure 2e–h. From the C 1*s* high‐resolution spectra (Figure 7c–e), it is evident that the C─C/C═C content in CHD‐CQDs_‐5‐HMF/FA‐10_ is the highest at 69.21%, followed by CHD‐CQDs_‐5‐HMF/LA‐10_ at 65.37%, and CHD‐CQDs_‐5‐HMF‐10_ at 57.79%. This further confirms that CHD‐CQDs_‐5‐HMF/FA‐10_ has the highest degree of graphitization, consistent with the Raman results. The O 1*s* high‐resolution spectra (Figure 7f–h) reveal that the O─C contents in CHD‐CQDs_‐5‐HMF‐10_, CHD‐CQDs_‐5‐HMF/FA‐10_, and CHD‐CQDs_‐5‐HMF/LA‐10_ are 46.98%, 63.30%, and 56.75%, respectively. This indicates that CHD‐CQDs_‐5‐HMF/FA‐10_ has the highest surface hydroxyl content. This observation may be attributed to the high O/C ratio in the carbon precursor used for CHD‐CQDs_‐5‐HMF/FA‐10_. Specifically, the atomic percentage of oxygen in FA is as high as 40%, whereas in 5‐HMF and LA, the oxygen atomic percentages are 29.41% and 18.75%, respectively. During the hydrothermal process, the chemical structure and properties of the precursors change, and oxygen may be retained in various forms or participate in the formation of products, thus affecting the O/C ratio of the CQDs. Therefore, under identical conditions, when 5‐HMF and FA (both with higher O/C ratio) are used as carbon sources, the O/C ratio in CHD‐CQDs_‐5‐HMF/FA‐10_ is higher than that in CHD‐CQDs_‐5‐HMF‐10_ and CHD‐CQDs_‐5‐HMF/LA‐10_, with most of the oxygen existing as ‐OH groups. The complete data for CHD‐CQDs_‐5‐HMF_, CHD‐CQDs_‐5‐HMF/FA_, and CHD‐CQDs_‐5‐HMF/LA_ are detailed in Table S4 and Figures S9 and S10 (Supporting Information). It can be observed that as the hydrothermal time increases, the O/C ratio of CHD‐CQDs gradually increases, and at a hydrothermal time of 10 h, the O/C ratio of all three CHD‐CQDs reaches its peak. As hydrothermal time increases, the oxygen‐to‐carbon ratio of CHD‐CQDs gradually rises, reaching a maximum at 10 h across all three types of CHD‐CQDs. This trend can be explained by the formation mechanism of biomass‐derived CQDs, which fundamentally differs from the direct polymerization/carbonization pathway of small‐molecule precursors. The process typically involves: decomposing biomass into small molecules (e.g., 5‐HMF, FA, LA) under high‐temperature and high‐pressure conditions; crosslinking and aggregating these molecules through esterification and aldol condensation; followed by carbonization and nucleation. Subsequently, residual molecules continuously deposit onto the carbon core to form clusters, followed by further carbonization to form shell structure of CQDs. The formation of the core‐shell structure of CQDs results from the continuous clustering and carbonization of small molecules. Throughout this process of CQDs formation, oxygen is introduced during cluster formation, first increasing the O/C ratio, while continuous carbonization further promotes intramolecular dehydration, reducing the oxygen content on the surface of CQDs. Therefore, the increase in O/C is observed prior to 10 h with small molecules continuously incorporated into growing CQDs. After 10 h, when small molecules are depleted, further dehydration and carbonization of the formed CQDs become more pronounced, leading to a gradual decrease in O/C ratio.

The fluorescence performance (fluorescence intensities and QYs in Table S1, Supporting Information) of CHD‐CQDs_‐5‐HMF_, CHD‐CQDs_‐5‐HMF/F_, CHD‐CQDs_‐5‐HMF/FA_, and CHD‐CQDs_‐5‐HMF/LA_ initially increases and then decreases as hydrothermal time extends. Among them, the fluorescence performance of CHD‐CQDs_‐5‐HMF/F_ is the lowest, while CHD‐CQDs_‐5‐HMF/FA_ and CHD‐CQDs_‐5‐HMF/LA_ exhibit better fluorescence properties. This correlates positively with 5‐HMF consumption: during the formation of CHD‐CQDs_‐5‐HMF/F_, 5‐HMF is consumed slowly, whereas during the formation of CHD‐CQDs_‐5‐HMF/FA_ and CHD‐CQDs_‐5‐HMF/LA_, it is consumed rapidly. When the hydrothermal time reaches 10 h, the fluorescence performance of CHD‐CQDs peaks. CHD‐CQDs_‐5‐HMF/FA‐10_ exhibit the highest fluorescence intensity reaching 345 424. Compared with CES‐CQDs_0_ (56 539) synthesized using untreated cellulose as carbon source, the fluorescence intensity of CHD‐CQDs_‐5‐HMF/FA‐10_ demonstrate a more than 5‐fold enhancement. Combined with the aforementioned XPS analysis, the CHD‐CQDs_‐5‐HMF/FA‐10_ with the highest O/C ratio demonstrate the most superior fluorescence performance.

Figure 7i shows the UV–vis spectra of CHD‐CQDs_‐5‐HMF‐10_, CHD‐CQDs_‐5‐HMF/FA‐10_, and CHD‐CQDs_‐5‐HMF/LA‐10_. In combination with Figure S11 (Supporting Information) (the UV–vis spectra of CHD‐CQDs_‐5‐HMF_, CHD‐CQDs_‐5‐HMF/F_, CHD‐CQDs_‐5‐HMF/FA_, and CHD‐CQDs_‐5‐HMF/LA_), it is evident that, compared to the UV–vis spectra of CES‐CQDs and CEM‐CQDs in Figure 4, the absorption peak position of CHD‐CQDs remains unchanged, indicating that under ultraviolet light excitation, CHD‐CQDs undergo *π*–π* transitions below 300 nm and n‐π* transitions at ≈330 nm. Figure 7j–l show the 3D fluorescence spectra of CHD‐CQDs_‐5‐HMF‐10_, CHD‐CQDs_‐5‐HMF/FA‐10_, and CHD‐CQDs_‐5‐HMF/LA‐10_, respectively. The optimal excitation wavelength is 350 nm, the optimal emission wavelength is 440 nm, and the samples appear blue under ultraviolet light (Figure 7i, inset). Compared to the fluorescence spectra of CES‐CQDs and CEM‐CQDs in Figure 4, the peaks of CHD‐CQDs in Figures S12–S15 (Supporting Information) (the 3D fluorescence spectra of CHD‐CQDs_‐5‐HMF_, CHD‐CQDs_‐5‐HMF/F_, CHD‐CQDs_‐5‐HMF/FA_, and CHD‐CQDs_‐5‐HMF/LA_) are sharper, and the FWHM is narrower, indicating that CHD‐CQDs exhibit higher fluorescence purity.^[^ ^36^, ^37^ ^]^

### 2.4 Formation Mechanism of Cellulose‐Derived CQDs

**Figure 8:**
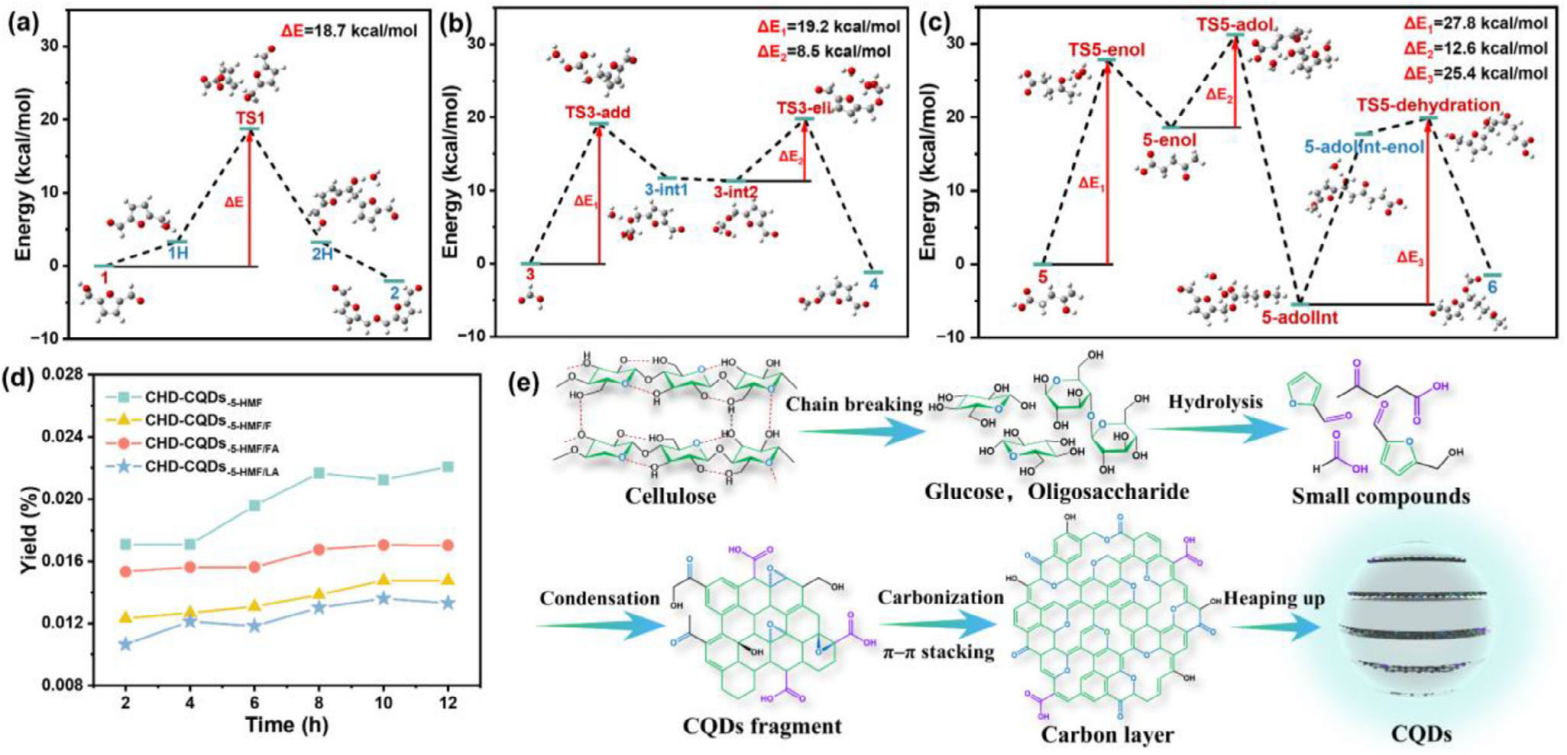
Potential energy distribution of reaction paths about CQDs formation: a) 5‐HMF self‐etherification reaction path; b) FA and 5‐HMF esterification reaction path; c) LA and 5‐HMF aldol condensation reaction path; d) CHD‐CQDs yield statistics. e) Inference of the formation mechanism of cellulose‐derived CQDs.

To more precisely determine the formation mechanism for cellulose‐derived CQDs and to elucidate the reasons behind their efficient synthesis and superior fluorescence properties, DFT was employed to calculate the kinetic data of the reaction. **Figure** **8**a–c illustrates the kinetic energy curves of the three reaction pathways involved in the formation of CQDs, along with the optimized geometric structures of the reactants, intermediates, transition states, and products. Figure 8a depicts the potential energy curve for the self‐etherification reaction of 5‐HMF. Structure 2 represents a small molecule compound generated by the etherification reaction of two 5‐HMF molecules. The entire reaction process requires overcoming a potential energy barrier of 18.7 kcal·mol^−1^. Figure 8b shows the potential energy curve for the esterification reaction between FA and 5‐HMF. Structure 4 is a small molecule compound produced by the esterification of one 5‐HMF molecule and one FA molecule. This reaction generates two potential energy peaks, with a total potential energy barrier of 27.7 kcal·mol^−1^ that must be overcome. Figure 8c presents the potential energy curve for the aldol condensation reaction between LA and 5‐HMF. Structure 6 is a small molecule compound formed by the aldol condensation of one 5‐HMF molecule and one LA molecule. This reaction produces three potential energy peaks, and the total potential energy barrier to be overcome is 65.8 kcal·mol^−1^. Analysis of the potential energy barriers for these three reaction pathways reveals that the self‐etherification of 5‐HMF has the lowest energy barrier and is the most facile reaction. This is followed by the esterification of 5‐HMF and FA, and finally the aldol condensation of 5‐HMF and LA. Correlation with the yield of CHD‐CQDs (Figure 8d) reveals that in a hydrothermal system containing solely 5‐HMF, the yield of CHD‐CQDs_‐5‐HMF_ reaches 2.21%. When employing 5‐HMF/FA and 5‐HMF/LA as precursors, the maximum yields of CHD‐CQDs_‐5‐HMF/FA_ and CHD‐CQDs_‐5‐HMF/LA_ are 1.70% and 1.36%, respectively. These findings indicate that a high concentration of 5‐HMF in the hydrothermal system facilitates the formation of CQDs, and the self‐etherification of 5‐HMF is more likely to yield CQDs efficiently. Additionally, compared to the 5‐HMF/LA system, the 5‐HMF/FA system is more conducive to the formation of CQDs. These results are consistent with the DFT analysis. Based on experimental data and kinetic analysis, the main reaction in the hydrothermal system is the self‐etherification of 5‐HMF, which is the critical pathway for preparing cellulose‐derived CQDs, followed by the esterification of 5‐HMF and FA, and the aldol condensation of 5‐HMF and LA. This provides a theoretical basis for regulating the cellulose carbon source to achieve high QY in cellulose‐derived CQDs.

Based on the above theoretical calculations and existing research results,^[^ ^38^, ^39^, ^40^ ^]^ it is proposed that the formation process of cellulose‐derived CQDs follows the mechanism shown in Figure 8e. At this relatively low temperature range (≤200 °C), the chemical bonds within the crystalline region of cellulose remain largely intact, resulting in minimal structural degradation.^[^ ^41^ ^]^ Cellulose dehydrates to produce polysaccharides and glucose. Simultaneously, the glucose begins to further degrade, generating intermediates such as 5‐HMF, FA, and LA. These intermediates undergo chemical reactions, primarily the self‐etherification of 5‐HMF and the esterification of 5‐HMF and FA, followed by dehydration polymerization to form polyester molecules. These molecules then rearrange to create a carbonized sheet structure. Finally, carbon sheets of varying sizes are oriented and combined through *π*–*π* stacking, ultimately forming spherical CQDs. During the preparation of CES‐CQDs, limited quantities of 5‐HMF, FA, and LA are generated through cellulose pyrolysis, leading to lower production yields and smaller particle sizes of CES‐CQDs. In contrast, during the preparation of CEM‐CQDs, a large amount of glucose and low molecular weight polysaccharides, which are cellulase hydrolysis products, participated in the reaction. These compounds subsequently undergo thermal decomposition, generating significant quantities of 5‐HMF, FA, and LA. The abundant small molecule fragments are conducive to extensive aggregation, making CEM‐CQDs have more complete carbon nuclei, larger particle size, and higher yield than CES‐CQDs. This process generates more reaction intermediates rich in oxygen‐containing functional groups during hydrothermal treatment. Consequently, the resulting CEM‐CQDs exhibit higher O/C ratios, enhanced fluorescence intensity, and superior QY compared to CES‐CQDs. The synthesis mechanism of CHD‐CQDs involves the reaction and polymerization of 5‐HMF, FA, and LA molecules under high‐temperature and high‐pressure conditions. Among all CQDs prepared in this study, CHD‐CQDs_‐5‐HMF/FA‐10_, synthesized using 5‐HMF and FA as carbon precursors at 200 °C for 10 h, demonstrate the highest O/C ratio and optimal fluorescence performance. In conclusion, for cellulose‐derived CQDs, an increase in O/C ratio has a significant enhancing effect on their QY.

### 2.5 Application of CQDs in Fe^3+^ Detection

**Figure 9:**
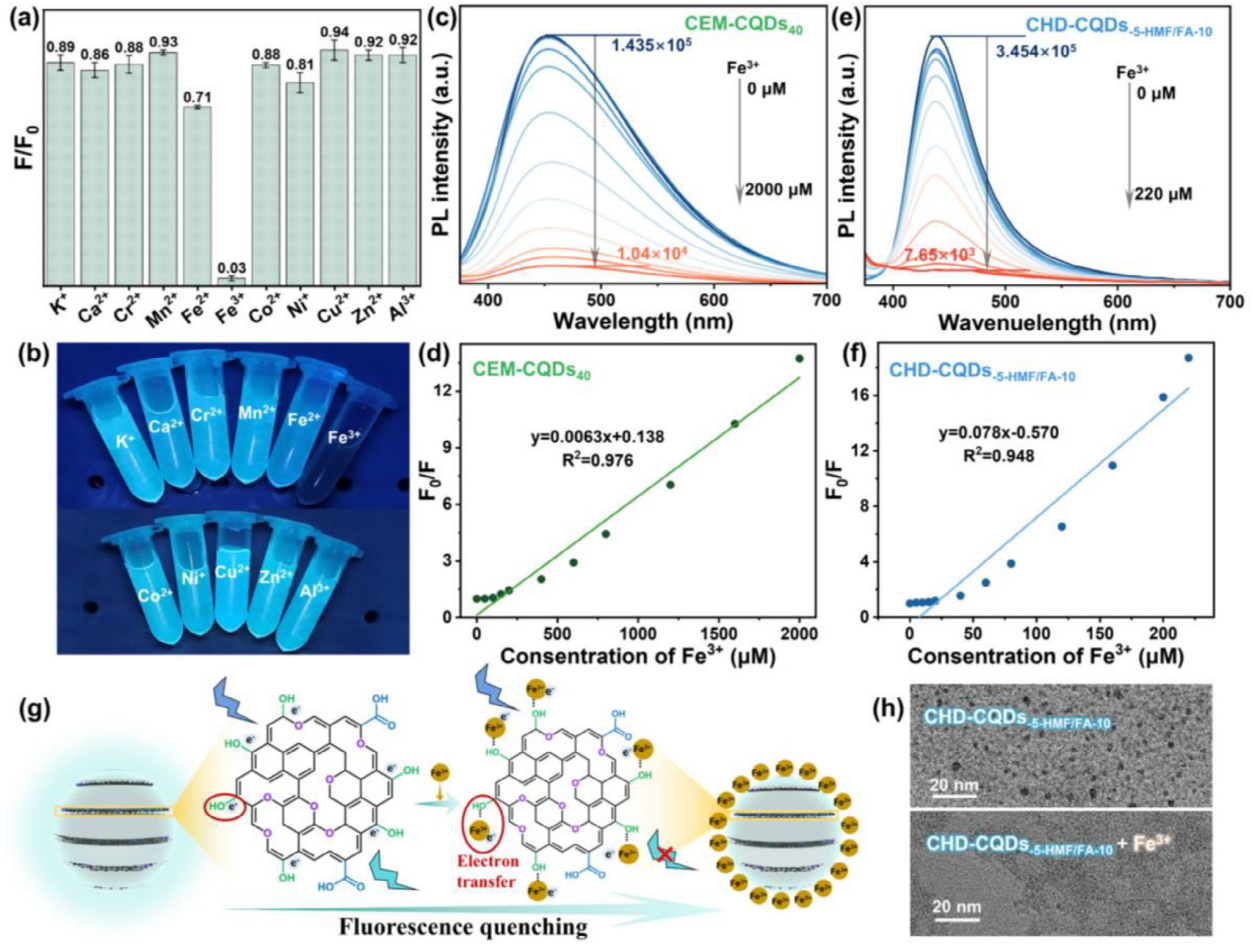
a) Effect of different metal ions on the fluorescence intensity ratio (F/F_0_) of CHD‐CQDs_‐5‐HMF/FA‐10_ and b) image of CQDs samples under 365 nm UV light. Fluorescence spectra of CQDs at different Fe^3+^ concentrations: c) CEM‐CQDs_40_ (Fe^3+^ concentration is 0–2 mm) and e) CHD‐CQDs_‐5‐HMF/FA‐10_ (Fe^3+^ concentration is 0–220 µm). F_0_/F is linearly related to Fe^3+^ concentration: d) CEM‐CQDs_40_ and f) CHD‐CQDs_‐5‐HMF/FA‐10_. g) Simulation of the fluorescence quenching process of CQDs combined with Fe^3+^ and h) TEM image of CQDs after fluorescence quenching.

In order to clarify the application potential of the prepared CEM‐CQDs_40_ and CHD‐CQDs_‐5‐HMF/FA‐10_ in the detection of metal ions in water, different metal ions (K^+^, Ca^2+^, Cr^3+^, Mn^2+^, Fe^2+^, Fe^3+^, Co^2+^, Ni^2+^, Cu^2+^, Zn^2+^, and Al^3+^) first were used to test their effects on the fluorescence intensity of CQDs at an excitation wavelength of 365 nm. The fluorescence intensity of CHD‐CQDs_‐5‐HMF/FA‐10_ and CEM‐CQDs_40_ is compared after adding different metal ions (F/F_0_, where F_0_ represents the fluorescence intensity when no metal ions are added), and the results are shown in **Figure** **9**a (CHD‐CQDs_‐5‐HMF/FA‐10_) and Figure S16 (Supporting Information) (CEM‐CQDs_40_). At the same time, Figure 9b is a sample image of the CQDs_‐5‐HMF/FA‐10_ solution under 365 nm ultraviolet light after adding different metal ions. It can be observed that Fe^3+^ has a significant quenching effect on the two CQDs compared with other metal ions, while the other metal ions have a smaller effect, indicating that both CQDs have the ability to selectively detect Fe^3+^.

In order to further break through the material performance boundaries in this field, this study focuses on optimizing the detection limit of CQDs, striving to achieve more sophisticated and efficient sensing technology. After adding different concentrations of Fe^3+^ to the CEM‐CQDs_40_ and CHD‐CQDs_‐5‐HMF/FA‐10_ solution, the change in its fluorescence intensity is investigated. It is found that in the Fe^3+^ concentration range of 0–2 mm, the fluorescence intensity of CEM‐CQDs_40_ slowly decreases from 1.435 × 10^5^ to 1.04 × 10^4^. Then, the Fe^3+^ concentration continues to increase, and the fluorescence intensity of CEM‐CQDs_40_ remains basically unchanged (Figure 9c). In the Fe^3+^ concentration range of 0–220 µm, the fluorescence intensity of CHD‐CQDs_‐5‐HMF/FA‐10_ slowly decreases from 3.454 × 10^5^ to 7.65 × 10^3^. When the Fe^3+^ concentration reaches 220 µm, the fluorescence intensity of CHD‐CQDs_‐5‐HMF/FA‐10_ remains nearly unchanged (Figure 9e).

The linear relationship between the fluorescence intensity change (F_0_/F) of CEM‐CQDs_40_ and CHD‐CQDs_‐5‐HMF/FA‐10_ and the Fe^3+^ ion concentration was fitted, respectively. The fitting formulas are shown in Figure 9d,f. The linear relationship between the fluorescence intensity of CEM‐CQDs_40_ and the Fe^3+^ ion (0–2000 µm) concentration is y = 0.0063x + 0.138; the linear relationship between the fluorescence intensity of CHD‐CQDs_‐5‐HMF/FA‐10_ and the Fe^3+^ ion (0–220 µm) concentration is y = 0.078x–0.570, and the correlation coefficients R of the two linear relationship are both greater than 0.9. It is calculated by Equation S5 (Supporting Information) that the limit of detection (*LOD*) of CEM‐CQDs_40_ for Fe^3+^ is 3.095 µm, the *LOD* of CHD‐CQDs_‐5‐HMF/FA‐10_ for Fe^3+^ is 0.296 µm, indicating that compared with CEM‐CQDs, CHD‐CQDs_‐5‐HMF/FA‐10_ has better selectivity in Fe^3+^ detection. At the same time, as shown in the statistical data of **Table** **1**, compared with the existing biomass‐based CQDs, CHD‐CQDs_‐5‐HMF/FA‐10_ shows a relatively superior ability in the detection of Fe^3+^.

**Table 1:** Detection performance of different biomass‐based CQDs for Fe^3+^.

Raw materials	Preparation method	Detection limit	References
Sweet potato	HTC	0.32 µm	[42]
Dwarfing banana peel	HTC	0.66 µm	[43]
Rose	HTC	0.959 µm	[44]
Bay laurel leaves	HTC	0.34 µm	[45]
Camellia husk	HTC	0.75 µm	[46]
5‐HMF and FA	HTC	0.296 µm	This experiment

The detection mechanism of Fe^3+^ is shown in Figure 9g. When CQDs encounter Fe^3+^, Fe^3+^ as the central atom shares electron pairs with the oxygen atoms in the hydroxyl groups on the surface of CQDs, and Fe^3+^ forms coordination bonds with the hydroxyl groups.^[^ ^47^ ^]^ In this process, electrons are transferred from the oxygen atoms in the hydroxyl groups to Fe^3+^, resulting in the capture of some electrons, and reducing the number of excited state electrons available for fluorescence emission, thereby causing fluorescence quenching of CQDs. However, compared to CEM‐CQDs_40_, CHD‐CQDs_‐5‐HMF/FA‐10_ exhibit a higher O/C ratio and a greater abundance of surface hydroxyl groups, facilitating stronger coordination between Fe^3+^ and the hydroxyl groups on the CHD‐CQDs. As a result, the fluorescence of CHD‐CQDs_‐5‐HMF/FA‐10_ is more readily quenched. The TEM comparison of Figure 9h also shows that after Fe^3+^ coordinates with CHD‐CQDs_‐5‐HMF/FA‐10_, its surface is almost completely covered by Fe^3+^, and the spherical nanoparticle structure of conventional CQDs is almost unobservable. These results show that at the same Fe^3+^ concentration, there are more hydroxyl sites on the surface of CHD‐CQDs_‐5‐HMF/FA‐10_ than that of CEM‐CQD_S40_, and the fluorescence induced by surface states is greatly affected by Fe^3+^, so that the detection effect of CEM‐CQD_S40_ on Fe^3+^ is more excellent.

## 3 Conclusion

This study presents an effective strategy for improving the fluorescence performance of cellulose‐derived CQDs by controlling the degradation forms (different molecular weights) of cellulose precursors. The results show that increasing the O/C ratio on the surface of CQDs (from 0.25 to 0.61) enhances the fluorescence intensity of cellulose‐derived CQDs by more than five‐fold and raises their QY by about seven‐fold. Among the different cellulose degradation products, CHD‐CQDs_‐5‐HMF/FA‐10_, synthesized from 5‐HMF and FA, exhibit the blue light emission with the highest fluorescence intensity, and the best performance for Fe^3^⁺ detection in water (0.296 µm). This study has elucidated the optimal hydrothermal synthesis route for cellulose‐derived CQDs: 5‐HMF acts as the key precursor, with its self‐etherification reaction playing a pivotal role, followed by the esterification of 5‐HMF with FA, and ultimately the aldol condensation of 5‐HMF with LA. This research not only provides a deep understanding of the synthesis mechanism of cellulose‐derived CQDs but also lays a theoretical foundation for their applications in metal ion detection, promoting further development in this field.

## 4 Experimental Section

### Enzymatic Hydrolysis of Cellulose

2% (w/v) cellulose and 15 FPU/g cellulase were added to a 500 mL conical flask, and the pH of the system was maintained at 4.8 with sodium citrate buffer. Enzymatic hydrolysis was carried out in an oscillator at 50 °C and 150 rpm. After the reaction, the enzyme was inactivated at high temperature, and the solid‐liquid mixture samples with enzymatic hydrolysis times of 0, 1, 5, 10, 20, and 40 min were collected for later use. The solids after enzymatic hydrolysis were named C_0_, C_1_, C_5_, C_10_, C_20_, and C_40_ according to the enzymatic hydrolysis time, and the DP of the solid residue after enzymatic hydrolysis was determined by the copper ethylenediamine method. The solid‐liquid mixture after enzymatic hydrolysis was named E_1_, E_5_, E_10_, E_20_, and E_40_ according to the enzymatic hydrolysis time.

### Preparation of CES‐CQDs

0.5 g of absolutely dry cellulose residues (C_0_, C_1_, C_5_, and C_40_), which had significant differences in DP, were weighed and mixed with 25 mL of deionized water. The mixture was placed in a 50 mL polytetrafluoroethylene‐lined reactor and heated at 200 °C for 12 h. After the reaction, the reactor was allowed to cool naturally to room temperature. The resulting product in the reaction kettle was filtered, and the supernatant liquid was collected. The supernatant was dialyzed (1000 Da), concentrated, and lyophilized to obtain solid CQDs, which were named CES‐CQDs_0_, CES‐CQDs_1_, CES‐CQDs_5_, and CES‐CQDs_40_, respectively.

### Preparation of CEM‐CQDs

25 mL E_1_, E_5_, and E_40_ were used as carbon sources, and CQDs were prepared by hydrothermal method. The reaction conditions were the same as above (preparation of CES‐CQDs). The prepared CQDs were named CEM‐CQDs_1_, CEM‐CQDs_5_, and CEM‐CQDs_40_, respectively.

### Preparation of CHD‐CQDs

1 M stock solutions of 5‐HMF, F, FA, and LA were prepared and mixed in a ratio of 0.5 M:0.5 M to obtain 5‐HMF/F, 5‐HMF/FA, and 5‐HMF/LA solutions. Then, 25 mL of each of the four solutions (5‐HMF, 5‐HMF/F, 5‐HMF/FA, and 5‐HMF/LA) was used as the carbon source to synthesize four CQDs under identical reaction conditions. These CQDs were designated as CHD‐CQDs_‐5‐HMF_, CHD‐CQDs_‐5‐HMF/F_, CHD‐CQDs_‐5‐HMF/FA_, and CHD‐CQDs_‐5‐HMF/LA_, respectively. The hydrothermal time was increased from 2 to 12 h, and the generation of CHD‐CQDs was detected every 2 h. The prepared CHD‐CQDs were named as follows: taking CHD‐CQDs_‐5‐HMF_ as an example, they were named CHD‐CQDs_‐5‐HMF‐2_, CHD‐CQDs_‐5‐HMF‐4_, CHD‐CQDs_‐5‐HMF‐6_, CHD‐CQDs_‐5‐HMF‐8_, CHD‐CQDs_‐5‐HMF‐10_, and CHD‐CQDs_‐5‐HMF‐12_, and the other three CHD‐CQDs were named in the same way.

### Metal Ion Sensing for CQDs

Common representative metal ions of different major groups in the periodic table (IA: K^+^; IIA: Ca^2+^; VIB: Cr^3+^; VIIB: Mn^2+^; VIII: Fe^2+^, Fe^3+^, Co^2+^, Ni^2+^; IB: Cu^2+^; IIB: Zn^2+^; IIIA: Al^3+^) were detected the fluorescence quenching effect of CQDs. The metal ion was diluted in 5 mL of phosphate buffer solution (PBS) at an appropriate concentration to minimize the effect of pH fluctuations caused by partial hydrolysis of the metal ions on the fluorescence properties. Then 5 mL of CQDs solution was added to the metal ion solution (the concentration of metal ions in the mixed solution was 1 mm, and the concentration of CQDs was 0.1 mg mL^−1^). The mixture solution was shaken at room temperature for 15 min. The fluorescence quenching properties of CQDs for metal ions were clarified by determining the change in fluorescence intensity of CQDs before and after mixing. Then, further experiments were carried out with a metal ion that had the best fluorescence quenching effect on CQDs to study the effect of metal ion concentration on the fluorescence intensity of CQDs. The linear relationship between the fluorescence intensity variation and metal ion concentration of CEM‐CQDs and CHD‐CQDs_‐5‐HMF/FA_ was analyzed to evaluate their effectiveness in metal ion detection.

## Conflict of Interest

The authors declare no conflict of interest.

## Supporting information



Supporting Information

## Data Availability

Research data are not shared.
