# Supplementary material for: O/C Ratio‐Driven Fluorescence Enhancement in Cellulose‐Derived Carbon Quantum Dots: Mechanistic Insights into Reaction Pathways
Source: Adv Sci (Weinh). 2025 Oct 6;12(47):e10577. doi: 10.1002/advs.202510577 (PMC12713033; doi:10.1002/advs.202510577)
Supplement: Supplementary file 1 — Supporting Information [file ADVS-12-e10577-s001.docx]

Supporting Information

**O/C Ratio-Driven Fluorescence Enhancement in Cellulose-Derived Carbon Quantum Dots: Mechanistic Insights into Reaction Pathways**

Yarong Shi, Siyu Zhao, Xiheng Kang, Xinrui Chen, Xue Ou, Mengnan Chong, Xueping Song*, Zhanying Zhang

*Y. Shi, S. Zhao, X. Kang, X. Chen, X. Ou, X. Song*

Guangxi Key Laboratory of Clean Pulp & Papermaking and Pollution Control, College of Light Industry and Food Engineering

Guangxi University

Nanning, 530004, PR China.

*E-mail: [sx_ping@gxu.edu.cn](mailto:sx_ping@gxu.edu.cn)

ORCID ID: The ORCID identification number for the corresponding author of this article can be found under <https://orcid.org/0000-0002-3373-0435>

*M. Chong*

Department of Chemical Engineering, Monash University Malaysia, Jalan Lagoon Selatan, Bandar Sunway, 47500 Selangor DE, Malaysia

*M. Chong*

Centre for Net-Zero Technology, Monash University Malaysia, Jalan Lagoon Selatan, Bandar Sunway, 47500 Selangor DE, Malaysia

*Z. Zhang*

School of Mechanical, Medical and Process Engineering, Centre for Agriculture and the Bioeconomy

Queensland University of Technology

Brisbane, Queensland 4000, Australia

**Reagents and materials**

Pulp cellulose fiber was obtained from a paper mill in Guangxi Province, China. Citric acid-sodium citrate buffer was purchased from Codow (Guangzhou, China). Copper (II) dihydroxide ethylenediamine solution was purchased from McLean Biochemical Technology Co (Shanghai, China). H_2_SO_4_ (95.0-98.0%) was purchased from Cologne Chemical Co (Chengdu, China). Cellulase (enzyme mixture), 5-HMF (>99%), F (>99%), FA (>99%), LA (>99%) and 11 kinds of metal salts (KCl, CaCl_2_, CrCl_3_, MnCl_2_·4H_2_O, FeCl_2_·4H_2_O, FeCl_3_, CoCl_2_·6H_2_O, Ni(NO_3_)·6H_2_O, CuSO_4_, ZnCl_2_, and AlCl_3_) were purchased from Aladdin Reagent, Co., Ltd. (Shanghai, China). The ultrapure water used in this experiment was obtained from Merck Milli-Q ultrapure water purification system.

**Testing and characterization**

High performance liquid chromatography (HPLC, AGILENT 1260 Infinity II LC, USA) was used to determine the glucose content in the enzymatic hydrolyzate system to measure the degree of hydrolysis at the corresponding stage. The cellulose powder samples before and after enzymatic hydrolysis and CQDs powder samples were tested using an X-ray diffractometer (Rigaku D/MAX 2500V, Japan), with a scanning range of 2θ=5°~80°. The elemental analyzer (EA, Elementar Vario EL cube, Germany) was used to determine the O/C ratio in the carbon precursor and CQDs, and the UV-visible spectrophotometer (UV-Vis, Hitachi U-4100 UV-Vis, Japan) was used to measure the UV-visible absorption spectra of the CQDs solution in the range of 200-800 nm. The chemical structure of CQDs was determined by Fourier transform infrared spectrometer (FTIR, TENSOR II, Bruker, Germany) in attenuated total reflectance (ATR) mode. The carbon, oxygen and other element contents and the surface functional group composition of CQDs were determined by X-ray photoelectron spectrometer (XPS, Thermo Fisher Scientific K-alpha+, USA). The microstructure of CQDs was observed using a transmission electron microscope (TEM, FEI TECNAI G2 F30, USA). The graphitization degree of CQDs was analyzed using a micro-Raman spectrometer (Raman), with a laser wavelength of 630 nm and a power of 0.10 mW. The fluorescence spectra (FLs) and PLQY of CQDs were measured using a fluorescence spectrometer (Hitachi RF-5301PC, Japan), and the widths of the excitation slit and the emission slit were both 3 nm when measuring FLs. The fluorescence lifetime (𝜏) of CQDs was measured at an excitation wavelength of 365 nm using a fluorescence spectrometer (FLS-1000, Edinburgh, UK). Becke's three-parameter exchange functional and Lee-Yang-Parr correlation functional (B3LYP), combined with Grimme's DFT-D3 (BJ) empirical discretization correction, were used to perform DFT calculations on the transition dipole moments and oscillator energies of different molecular models in the Gaussian 09 program with the 6-31G* basis set ^[1]^. The formation mechanism of CQDs was calculated using the B3LYP functional combined with the 6-311g* basis set, and the SMD solvation model was used to consider the solvation effect of water.

**Supplementary results**

From the analysis results of elemental analysis (EA) in **Figure S1(a)**, it can be seen that as the degree of enzymatic hydrolysis increases, the oxygen content of C_0_, C_1_, C_5_, and C_40_ is 49.39%, 49.97%, 50.07%, and 50.11%, respectively. The oxygen content of E_1_, E_5_, and E_40_ is 50.07%, 50.41%, and 50.86%, respectively, showing minimal overall variation. **Figure S1(b)** shows the XRD spectra of the residual cellulose solid after enzymatic hydrolysis. The diffraction peaks at 2θ = 15.6° and 22.8°correspond to the (110) and (200) characteristic crystal planes of cellulose I, respectively.^[2]^

The crystallinity indexes (*CrI*) of cellulose C_0_, C_1_, C_5_, and C_40_ are 40.46%, 52.69%, 61.98%, and 69.92%, respectively, and increase with the extension of enzyme treatment time. This indicates that as the enzymatic hydrolysis time increases, the amorphous region of cellulose is destroyed, increasing the proportion of the crystalline region.^[3]^ In **Figure S1(c)**, FTIR analysis of the cellulose residual solids (C_0_, C_1_, C_5_, and C_40_) with significant changes in DP after enzymatic hydrolysis reveals the presence of -OH groups (3250-3500 cm⁻¹), methyl and methylene groups (2900 cm⁻¹), -C₆H₁₀O₅- groups on the cellulose molecular chain (1428 cm⁻¹), and hydrogen bonds at the carbon 2 or carbon 3 positions on the cellulose sugar ring (1202 cm⁻¹).^[4,5]^ The peak positions of the four enzymatically hydrolyzed celluloses remain unchanged. Since compared to the precursors of CES-CQDs (C_0_, C_1_, C_5_, and C_40_), the precursors of CEM-CQDs (E_1_, E_5_, and E_40_) contain not only cellulose with different DP, but also the enzymatic solution after cellulolytic digestion. Thus, the differences among the seven precursors are mainly in the DP of cellulose and the presence of glucose and small molecular weight polysaccharides.

XPS analysis was performed on the residual cellulose solids (C_0_, C_1_, C_5_, and C_40_), which exhibited significant changes in DP after enzymatic hydrolysis. **Figure S1(d)** is the full XPS spectra of cellulose. The two strong peaks at 284.8 and 533.5 eV correspond to C 1*s* and O 1*s*, respectively, indicating that the cellulose sample consists entirely of C and O elements. The atomic proportion of C is between 60% and 65%, while the atomic proportion of O ranges from 35% to 40%. **Figure S2(a)** shows the high-resolution C 1*s* energy spectra of four cellulose samples. The figure reveals three distinct peaks. The peak at 284.8 eV corresponds to C–C bonds in the basic structural unit of cellulose; the peak at 286.2 eV corresponds to C–O bonds; and the peak at 288.7 eV corresponds to C=O bonds of the aldehyde group in the glucose at the end of the cellulose chain.^[6]^ From the C 1*s* high-resolution energy spectra, it can be observed that as the enzyme treatment time increases, the content of C=O bonds gradually increases, with the proportion rising from 14.37% (C_0_ sample) to 21.62% (C_40_ sample). This further indicates that the number of exposed aldehyde groups in the terminal glucose of the cellulose sample increases. **Figure S2(b)** shows the O 1*s* high-resolution spectra of the cellulose sample. Two distinct peaks are observed in the spectra. The peak at 532.5 eV corresponds to the C=O bonds of the aldehyde group in the glucose unit at the end of the cellulose chain, while the peak at 533.3 eV is assigned to the C–O bonds in the phenolic hydroxyl group and the ether bond.^[7]^ From the O 1*s* high-resolution energy spectra, it is observed that as the enzyme treatment time increases, the C=O content in the four cellulose samples rises from 33.69% (C_0_ sample) to 47.53% (C_40_ sample), further indicating an increase in the exposed aldehyde groups in the terminal glucose of the cellulose samples. The XPS results indicate that the type of surface functional groups on cellulose samples remains consistent regardless of the degree of enzymatic hydrolysis, although there are variations in their content. These differences in functional group content directly influence the structure of the resulting CQDs, which in turn affects their properties.


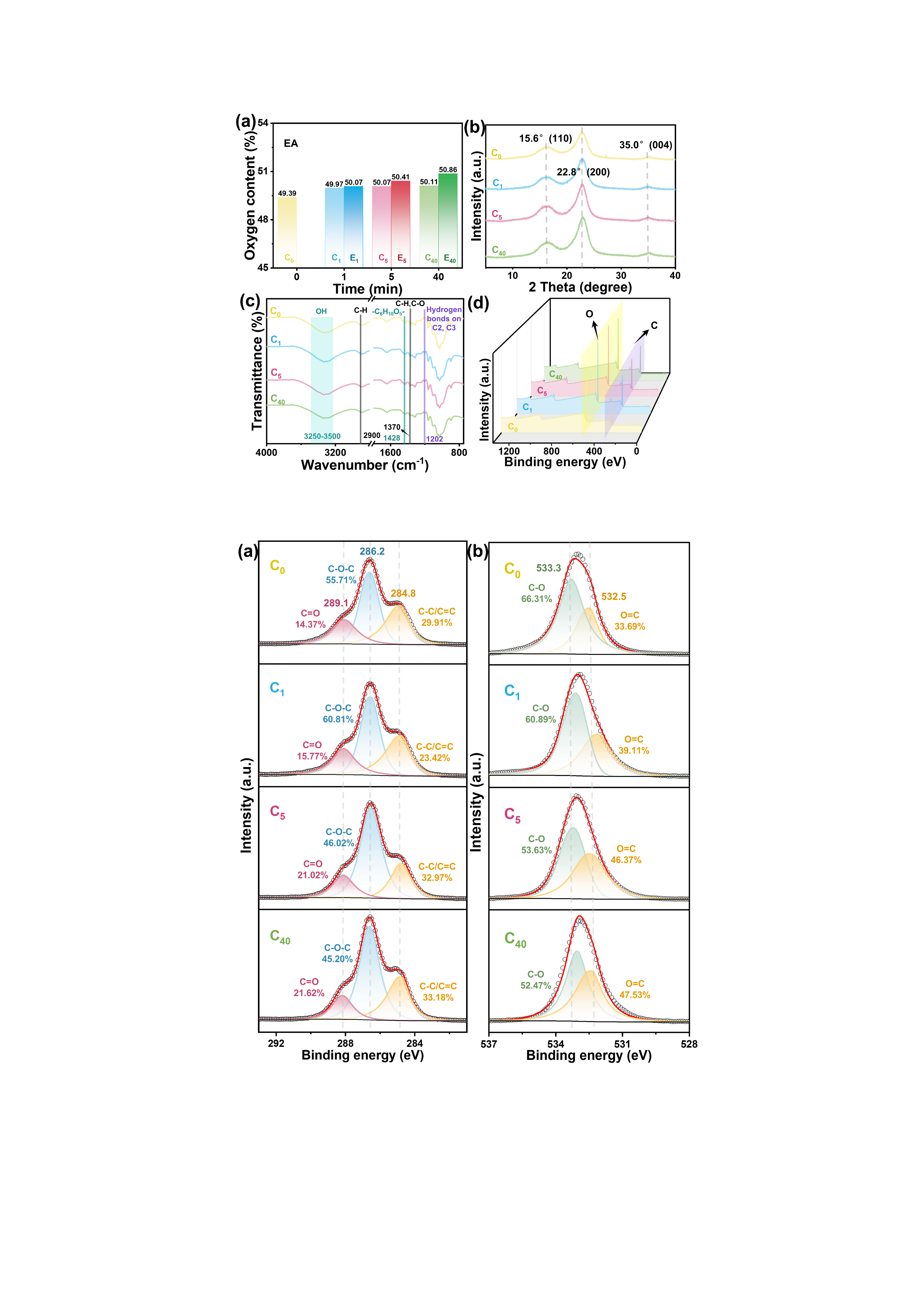


**Figure S1** Analysis of cellulose enzymatic hydrolysis products. (a) EA(O) of carbon precursor. (b) XRD spectra, (c) FT-IR spectra, (d) XPS full spectra


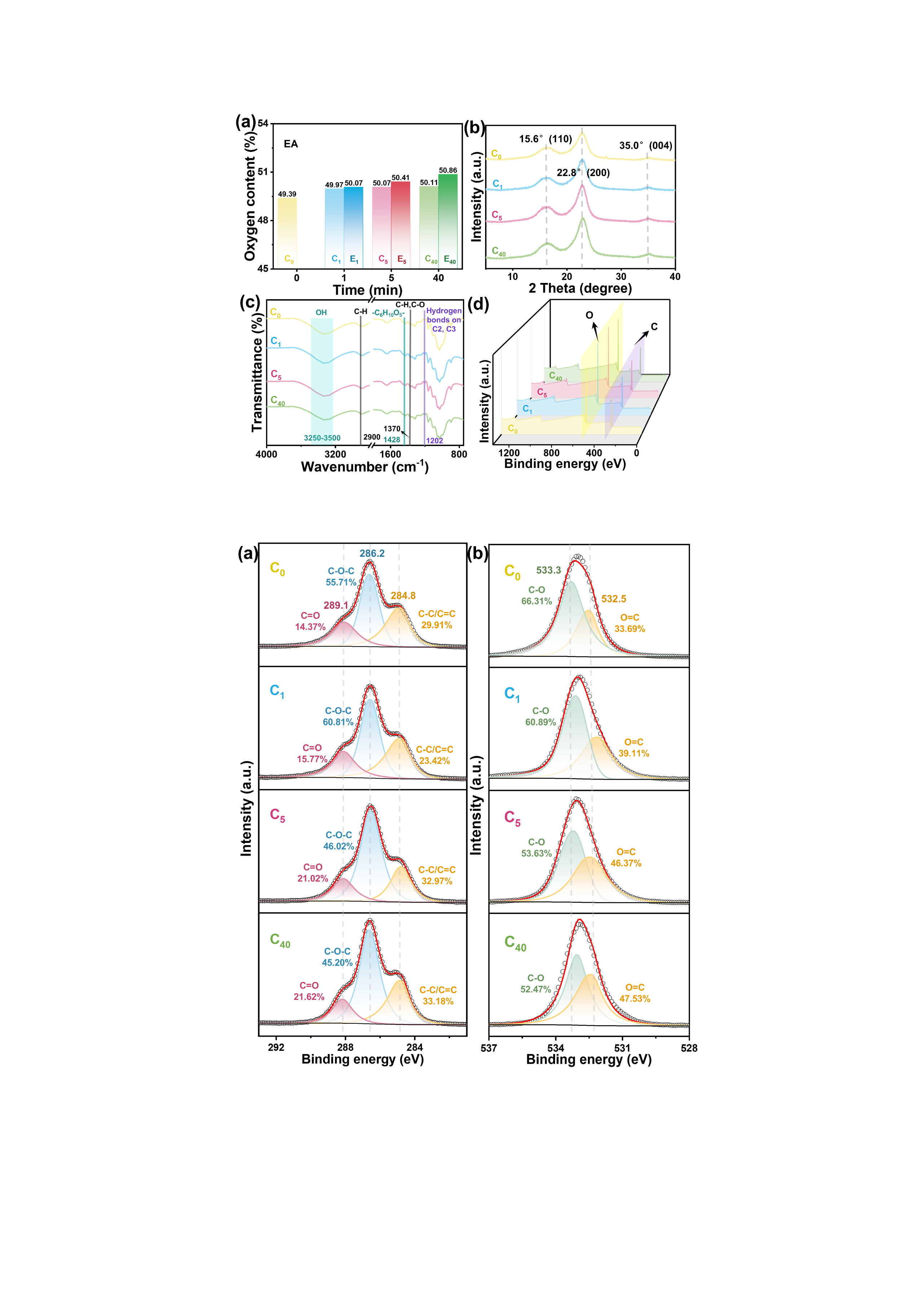


**Figure S2** High-resolution spectra of cellulose enzymatic hydrolysis products. (a) C 1*s* and (b) O 1*s*

The cellulose crystallinity index (*CrI*) is calculated using **Equation S1**:

$CrI =\frac{（I_{200}-I_{110}）}{I_{110}} \times100\%$ （S1）

Where: *I_200_* represents the peak intensity of the cellulose (200) crystal plane (2θ=22.8°); *I_am_* represents the peak intensity of the amorphous region (2θ=15.6°) obtained by fitting.


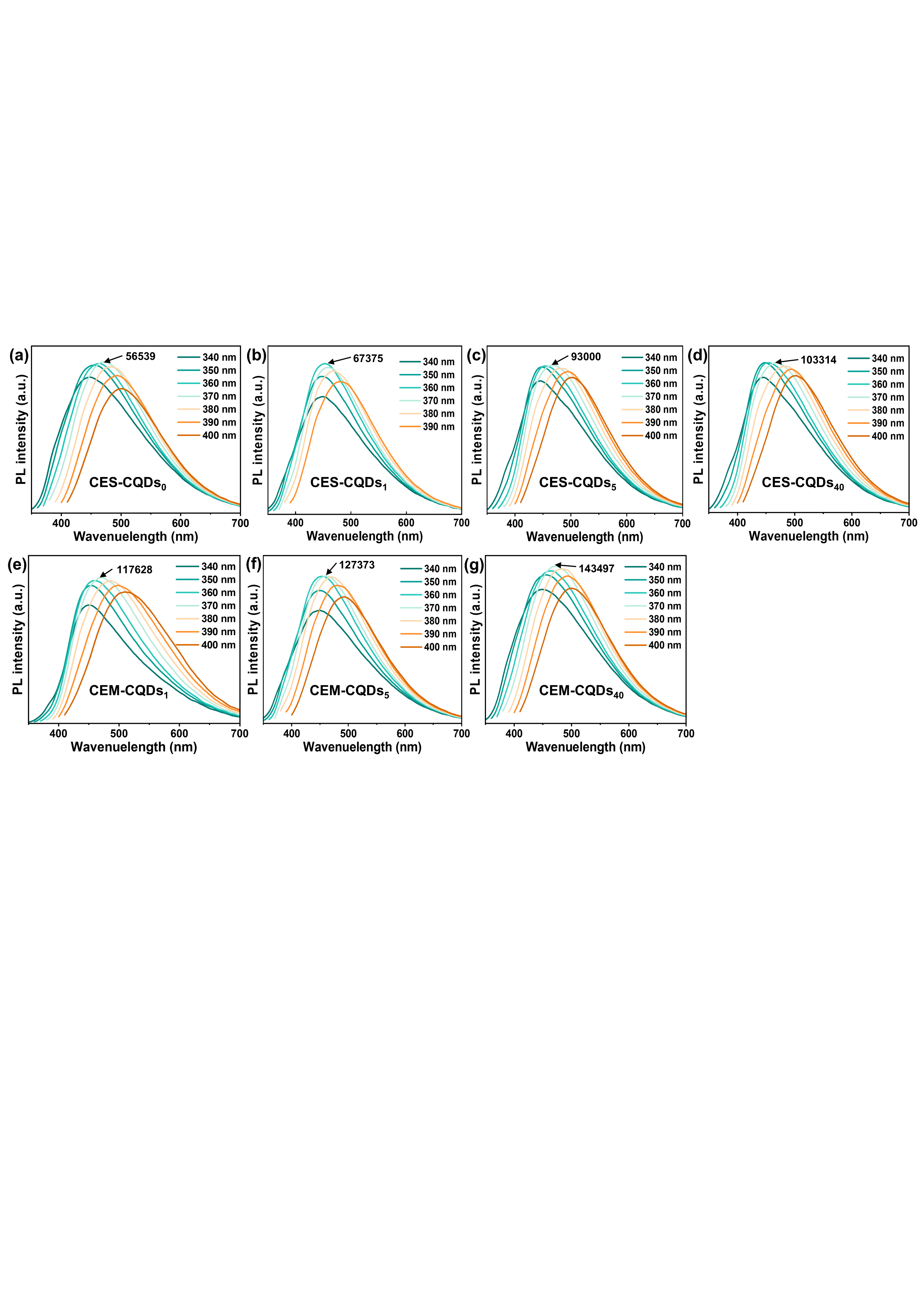


**Figure S3** 2D fluorescence spectra for all CES-CQDs and CEM-CQDs. (a) CES-CQDs_0_; (b) CES-CQDs_1_; (c) CES-CQDs_5_; (d) CES-CQDs_40_; (e) CEM-CQDs_1_; (f) CEM-CQDs_5_ and (g) CEM-CQDs_40_

**Table S****1** The PLQYs and fluorescence intensity (FL) summary of all CQDs

| Sample | PLQY | FL |
| --- | --- | --- |
| CES-CQDs_0_ | 0.70% | 56 539 |
| CES-CQDs_1_ | 0.71% | 67 375 |
| CES-CQDs_5_ | 0.99% | 93 000 |
| CES-CQDs_40_ | 1.87% | 103 314 |
| CEM-CQDs_1_ | 1.98% | 117 628 |
| CEM-CQDs_5_ | 2.51% | 127 373 |
| CEM-CQDs_40_ | 2.77% | 143 497 |
| CHD-CQDs_-5-HMF-2_ | 0.08% | 19 678 |
| CHD-CQDs_-5-HMF-4_ | 0.20% | 58 892 |
| CHD-CQDs_-5-HMF-6_ | 0.70% | 124 433 |
| CHD-CQDs_-5-HMF-8_ | 1.24% | 175 890 |
| CHD-CQDs_-5-HMF-10_ | 1.75% | 196 378 |
| CHD-CQDs_-5-HMF-12_ | 1.72% | 196 435 |
| CHD-CQDs_-5-HMF/F-2_ | 0.04% | 31 841 |
| CHD-CQDs_-5-HMF/F-4_ | 0.10% | 43 052 |
| CHD-CQDs_-5-HMF/F-6_ | 0.43% | 81 136 |
| CHD-CQDs_-5-HMF/F-8_ | 0.40% | 89 033 |
| CHD-CQDs_-5-HMF/F-10_ | 0.11% | 89 981 |
| CHD-CQDs_-5-HMF/F-12_ | 0.11% | 57 843 |
| CHD-CQDs_-5-HMF/FA-2_ | 0.50% | 45 539 |
| CHD-CQDs_-5-HMF/FA-4_ | 1.14% | 121 768 |
| CHD-CQDs_-5-HMF/FA-6_ | 1.31% | 142 774 |
| CHD-CQDs_-5-HMF/FA-8_ | 3.62% | 235 300 |
| **CHD-CQDs_-5-HMF/FA-10_** | **4.87%** | **345 424** |
| CHD-CQDs_-5-HMF/FA-12_ | 4.27% | 299 107 |
| CHD-CQDs_-5-HMF/LA-2_ | 0.23% | 24 759 |
| CHD-CQDs_-5-HMF/LA-4_ | 0.61% | 132 774 |
| CHD-CQDs_-5-HMF/LA-6_ | 2.20% | 163 836 |
| CHD-CQDs_-5-HMF/LA-8_ | 3.25% | 317 166 |
| CHD-CQDs_-5-HMF/LA-10_ | 4.28% | 341 709 |
| CHD-CQDs_-5-HMF/LA-12_ | 3.93% | 317 014 |

**Table S2** Summary of CQDs fluorescence lifetime data

| Sample | 𝜏_1_（ns） | A_1_（%） | 𝜏_2_（ns） | A_2_（%） | 𝜏_avg_（ns） |
| --- | --- | --- | --- | --- | --- |
| CES-CQDs_1_ | 1.28 | 60.13 | 4.89 | 39.87 | 2.66 |
| CES-CQDs_40_ | 1.32 | 58.49 | 5.03 | 41.51 | 2.86 |
| CEM-CQDs_1_ | 1.52 | 24.74 | 5.36 | 75.26 | 4.41 |
| CEM-CQDs_40_ | 1.58 | 23.62 | 5.39 | 76.38 | 4.49 |

The PL fluorescence decay curves of CQDs were fitted using **Equation S2**, and the fluorescence lifetime fitting curves of the four CQDs were obtained.

$I\left( t \right)=B_{1}exp\left( \frac{-t}{\tau_{1}} \right)+B_{2}exp\left( \frac{-t}{\tau_{2}} \right)$ （S2）

Among them, *B_1_* and *B_2_* are exponential factors; *𝜏_1_* and *𝜏_2_* are fitted life.

The PL decay curves of the four CQDs were fitted with a double exponential function (𝜏_1_ and 𝜏_2_), which was attributed to the two PL centers in the four CQDs^[8,9]^. According to the fitting parameters of the two decay curves, it can be seen that the four CQDs have two fluorescence lifetime decay channels, namely, a shorter lifetime (𝜏_1_: 0.87 ns, 1.32 ns, 1.52 ns and 1.58 ns) and a longer lifetime (𝜏_2_: 5.36 ns, 5.03 ns, 5.36 ns and 5.39 ns). This suggests that there are two competing decay pathways for exciton deactivation, in which the former PL center is associated with the radiative recombination of carbon core states (carbon nuclei with sp^2^ hybridized domains) where electrons are excited via π–π* transitions. The latter is related to the recombination process of surface states (containing O functional groups), where electrons are excited via n–π* transitions. In Table S1, A_1_ (%) and A_2_ (%) are calculated by weighting the exponential factors B_1_ and B_2_, representing the contributions of the fluorescence lifetimes 𝜏_1_ and 𝜏_2_, respectively. The average lifetime of CQDs is calculated by **Equation S3**:

$\tau_{avg}=\frac{B_{1}\tau_{1}^{2}+B_{2}\tau_{2}^{2}}{B_{1}\tau_{1}+B_{2}\tau_{2}}$ （S3）

The average consumption rate of 5-HMF is calculated using **Equation S4**：

$\text{R}\text{avg}\text{=}\frac{1}{12} \times\sum\frac{M_{i}-M_{i+2}}{M_{i}}$ （S4）

Where: *R_avg_* represents the average consumption rate of 5-HMF; *M_i_* represents the relative content of 5-HMF in the reaction system at the *i*-th hour (i=2, 4, 6, 8, 10)

**Table S3** Comparison of PLQYs in different biomass-based CQDs

| Raw materials | Preparation method | PLQY | References |
| --- | --- | --- | --- |
| EHL | HTC (220℃, 12 h) | 0.8% | ^[10]^ |
| Soybean peels | HTC (200℃, 2 h) | 2.5% | ^[11]^ |
| Crop wastes | HTC (160℃, 6 h) | 3.5% | ^[12]^ |
| Cassava stem | HTC (220℃, 10 h) | 2.24% | ^[13]^ |
| Cellulose | HTC (180℃, 3 h) | 2.2% | ^[14]^ |
| 5-HMF and FA | HTC (200℃, 12 h) | 4.87% | **This experiment** |

It can be found in **Table S3** that, compared with other biomass-based CQDs mainly composed of lignocellulose, the PLQYs of CHD-CQDs in this experiment have greater advantages. Building upon the established fluorescence characterization of CES-CQDs and CEM-CQDs, the experimental results substantiate that the degradation pretreatment effectively optimizes the luminescent characteristics of cellulose-based CQDs, with observable improvements in PLQY.

**Table S4** Summary of XPS peak data of CHD-CQDs

| Samples | O/C | C-C/C=C (%) | C-O-C (%) | O-C=O (%) | O-C (%) | O=C (%) |
| --- | --- | --- | --- | --- | --- | --- |
| CHD-CQDs_-5-HMF-2_ | 0.36 | 46.83 | 34.08 | 19.09 | 40.11 | 59.89 |
| CHD-CQDs_-5-HMF-6_ | 0.41 | 56.53 | 30.51 | 12.96 | 42.84 | 57.16 |
| CHD-CQDs_-5-HMF-10_ | 0.44 | 57.79 | 24.75 | 17.46 | 46.98 | 53.02 |
| CHD-CQDs_-5-HMF-12_ | 0.43 | 58.08 | 24.88 | 17.04 | 48.57 | 51.43 |
| CHD-CQDs_-5-HMF/FA-2_ | 0.33 | 49.72 | 31.49 | 18.79 | 32.39 | 67.61 |
| CHD-CQDs_-5-HMF/FA-6_ | 0.45 | 54.09 | 24.31 | 21.60 | 43.96 | 56.04 |
| CHD-CQDs_-5-HMF/FA-10_ | 0.61 | 69.21 | 19.93 | 10.86 | 63.30 | 36.70 |
| CHD-CQDs_-5-HMF/FA-12_ | 0.52 | 62.36 | 27.58 | 10.05 | 46.86 | 53.14 |
| CHD-CQDs_-5-HMF/LA-2_ | 0.40 | 43.38 | 31.65 | 24.97 | 32.15 | 67.85 |
| CHD-CQDs_-5-HMF/LA-6_ | 0.44 | 52.45 | 43.72 | 3.82 | 46.09 | 53.91 |
| CHD-CQDs_-5-HMF/LA-10_ | 0.56 | 68.92 | 22.01 | 9.07 | 56.75 | 43.25 |
| CHD-CQDs_-5-HMF/LA-12_ | 0.53 | 65.37 | 29.31 | 5.16 | 51.76 | 48.24 |

The lowest concentration of Fe^3+^ that could be detected was calculated using **Equation S5**.

$LOD= \frac{K \times S}{m}$ （S5）

Among them, *K* represents the confidence ratio, usually *K*=3; *S* represents the overall standard deviation of the sample blank; and *m* represents the linear slope of sample detection within a certain concentration range.


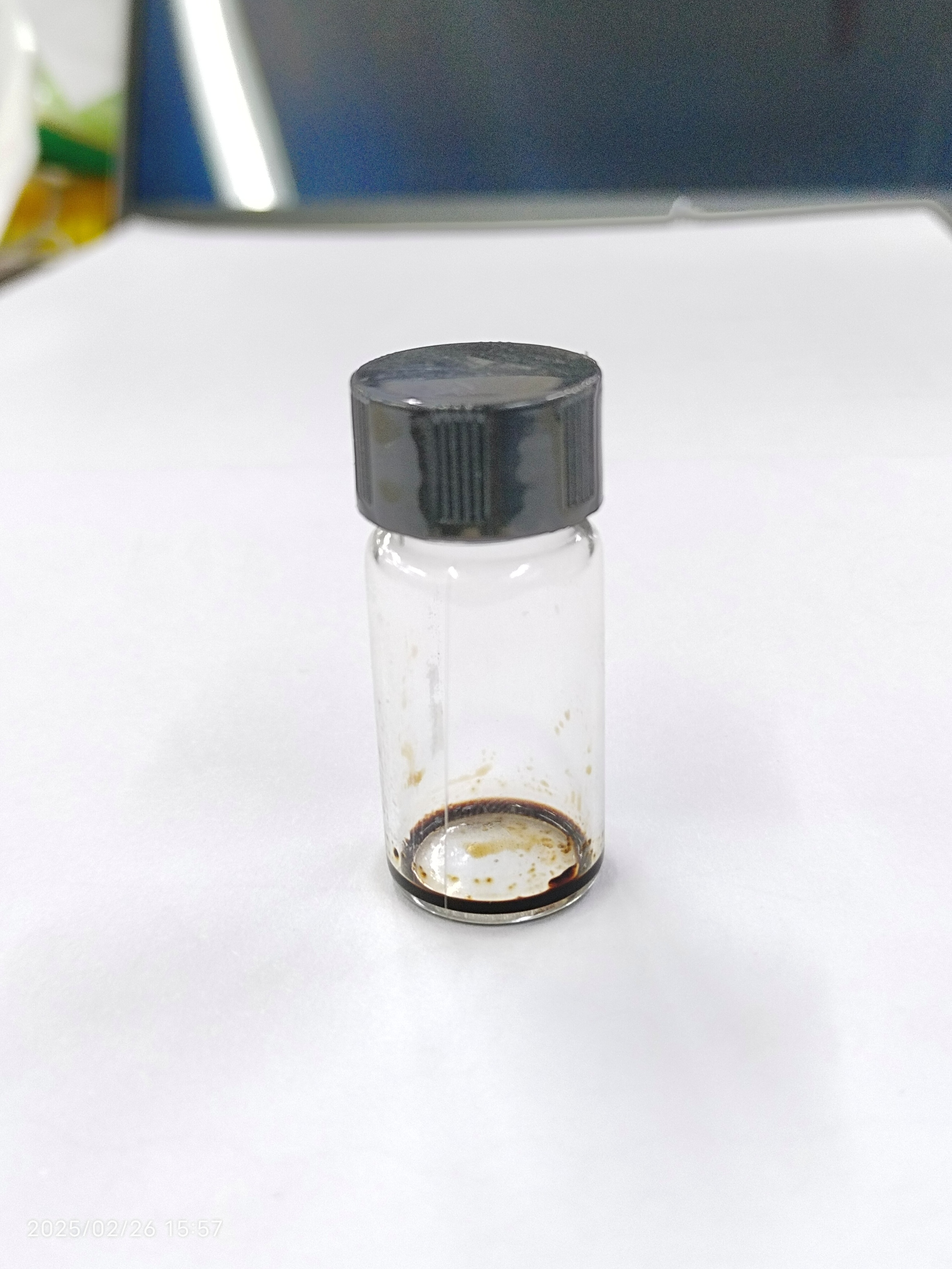


**Figure S4** CHD-CQDs_-F_


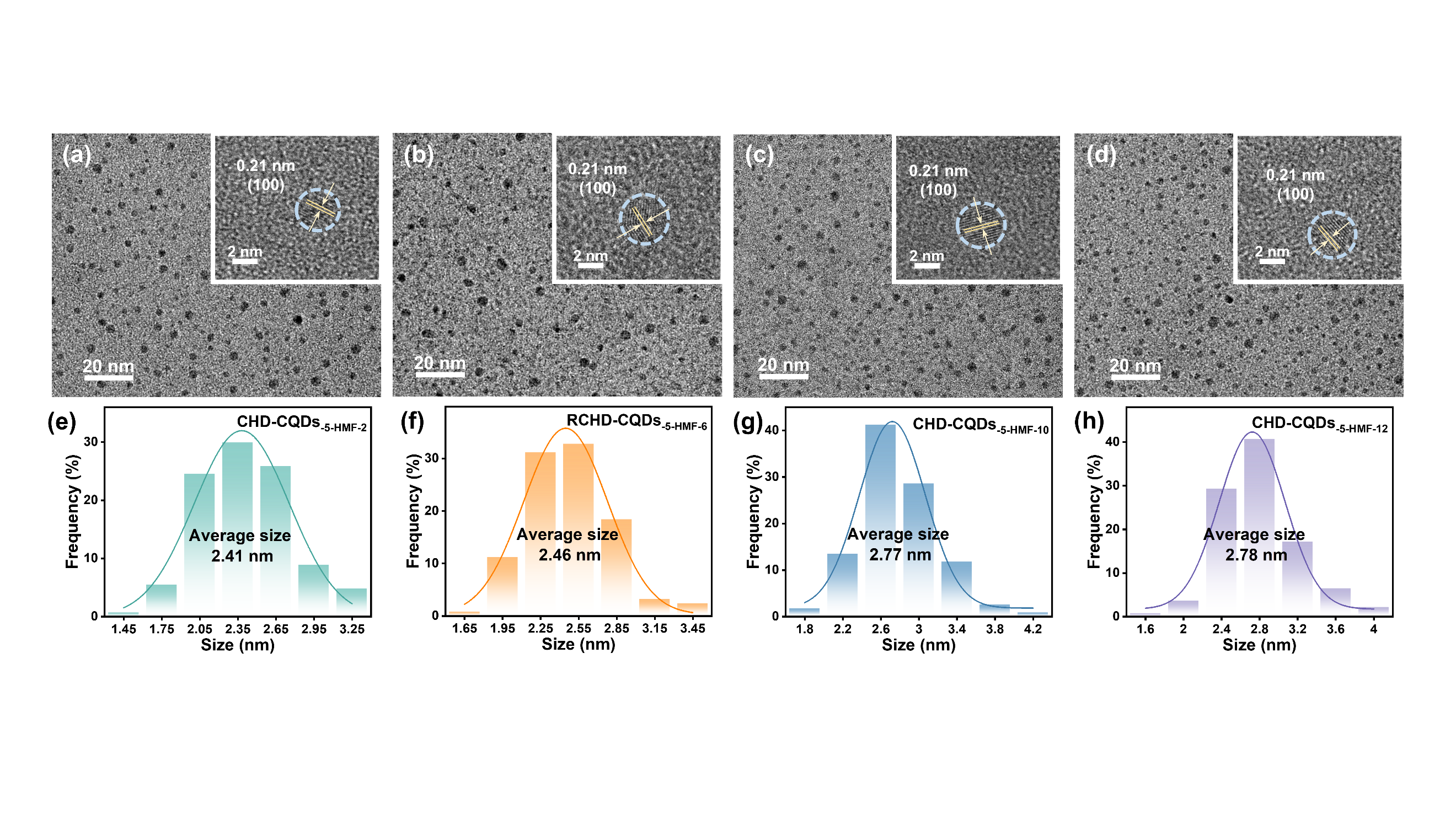


**Figure S5** TEM images of CHD-CQDs_-5-HMF_: (a) CHD-CQDs_-5-HMF-2_, (b) CHD-CQDs_-5-HMF-6_, (c) CHD-CQDs_-5-HMF-10_, and (d) CHD-CQDs_-5-HMF-12_; particle size distribution of CHD-CQDs_-5-HMF_: (e) CHD-CQDs_-5-HMF-2_, (f) CHD-CQDs_-5-HMF-6_, (g) CHD-CQDs_-5-HMF-10_, and (h) CHD-CQDs_-5-HMF-12_, (e) CHD-CQDs_-5-HMF-10_, (f) CHD-CQDs_-5-HMF-12_


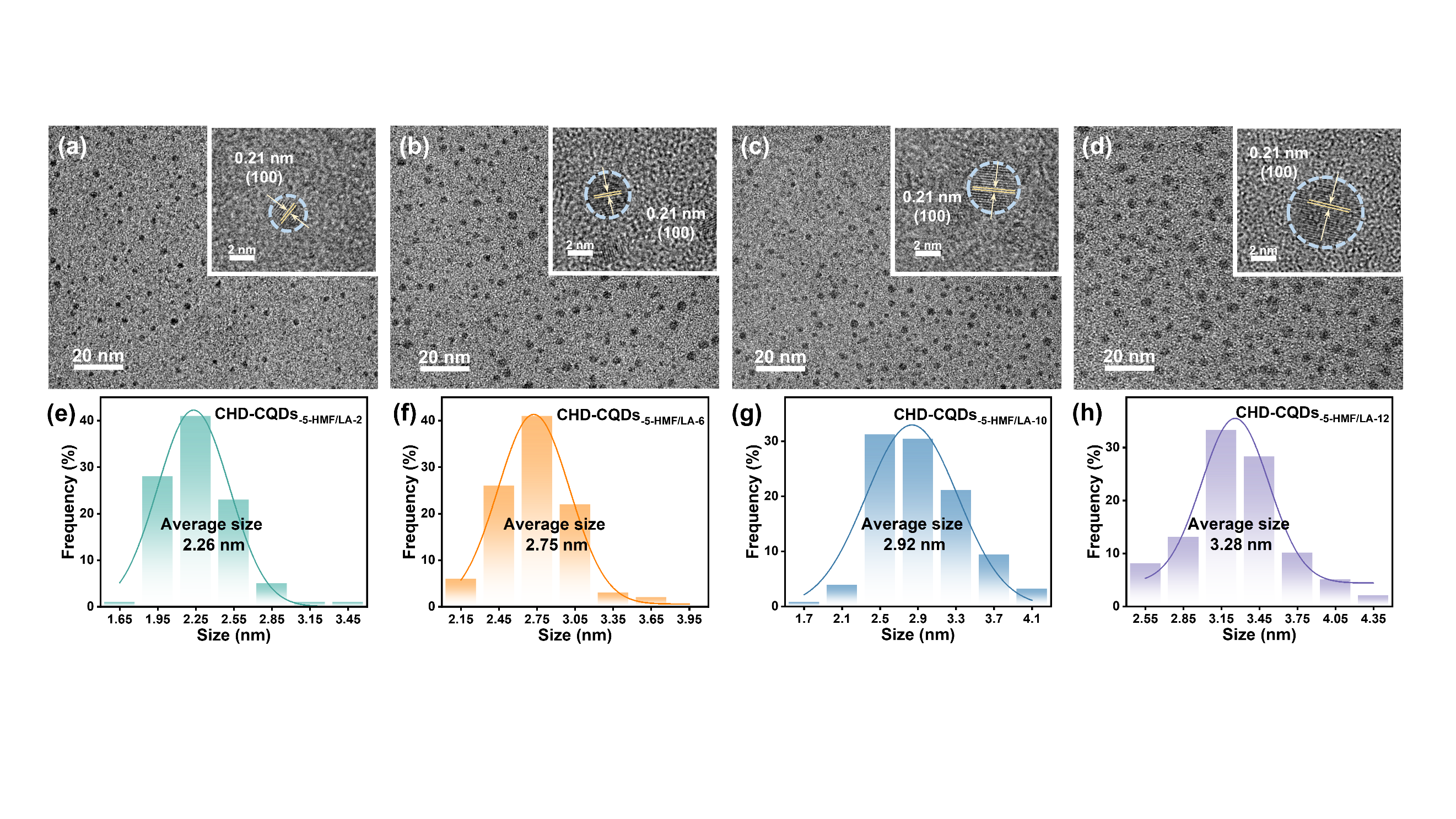


**Figure S6** TEM images of CHD-CQDs_-5-HMF/LA_: (a) CHD-CQDs_-5-HMF/LA-2_, (b) CHD-CQDs_-5-HMF/LA-6_, (c) CHD-CQDs_-5-HMF/LA-10_, and (d) CHD-CQDs_-5-HMF/LA-12_; particle size distribution of CHD-CQDs_-5-HMF/LA_: (e) CHD-CQDs_-5-HMF/LA-2_, (f) CHD-CQDs_-5-HMF/LA-6_, (g) CHD-CQDs_-5-HMF/LA-10_, and (h) CHD-CQDs_-5-HMF/LA-12_


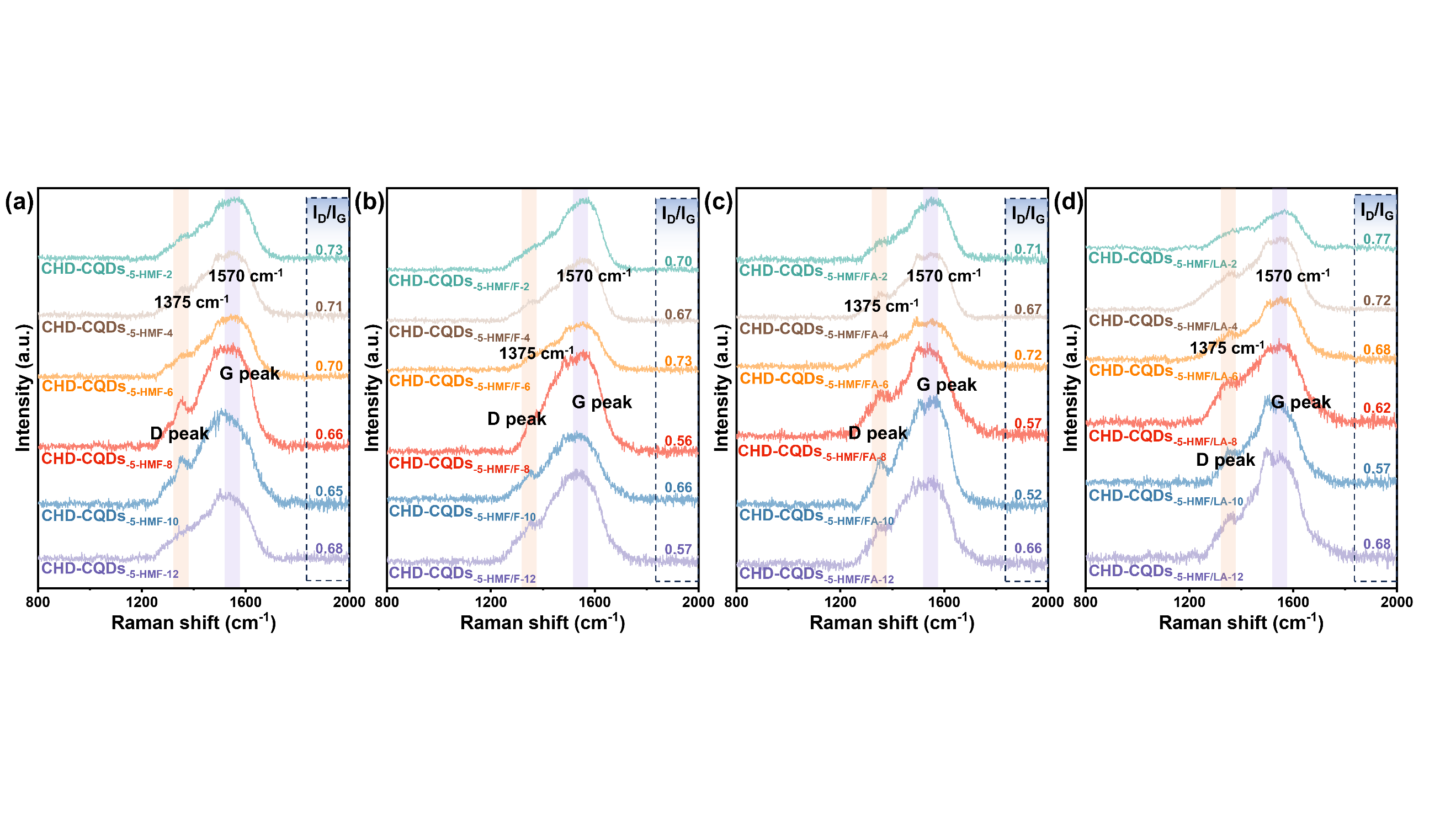


**Figure S7** Raman spectra of CHD-CQDs: (a) CHD-CQDs_-5-HMF_, (b) CHD-CQDs_-5-HMF/F_, (c) CHD-CQDs_-5-HMF/FA_, and (d) CHD-CQDs_-5-HMF/LA_


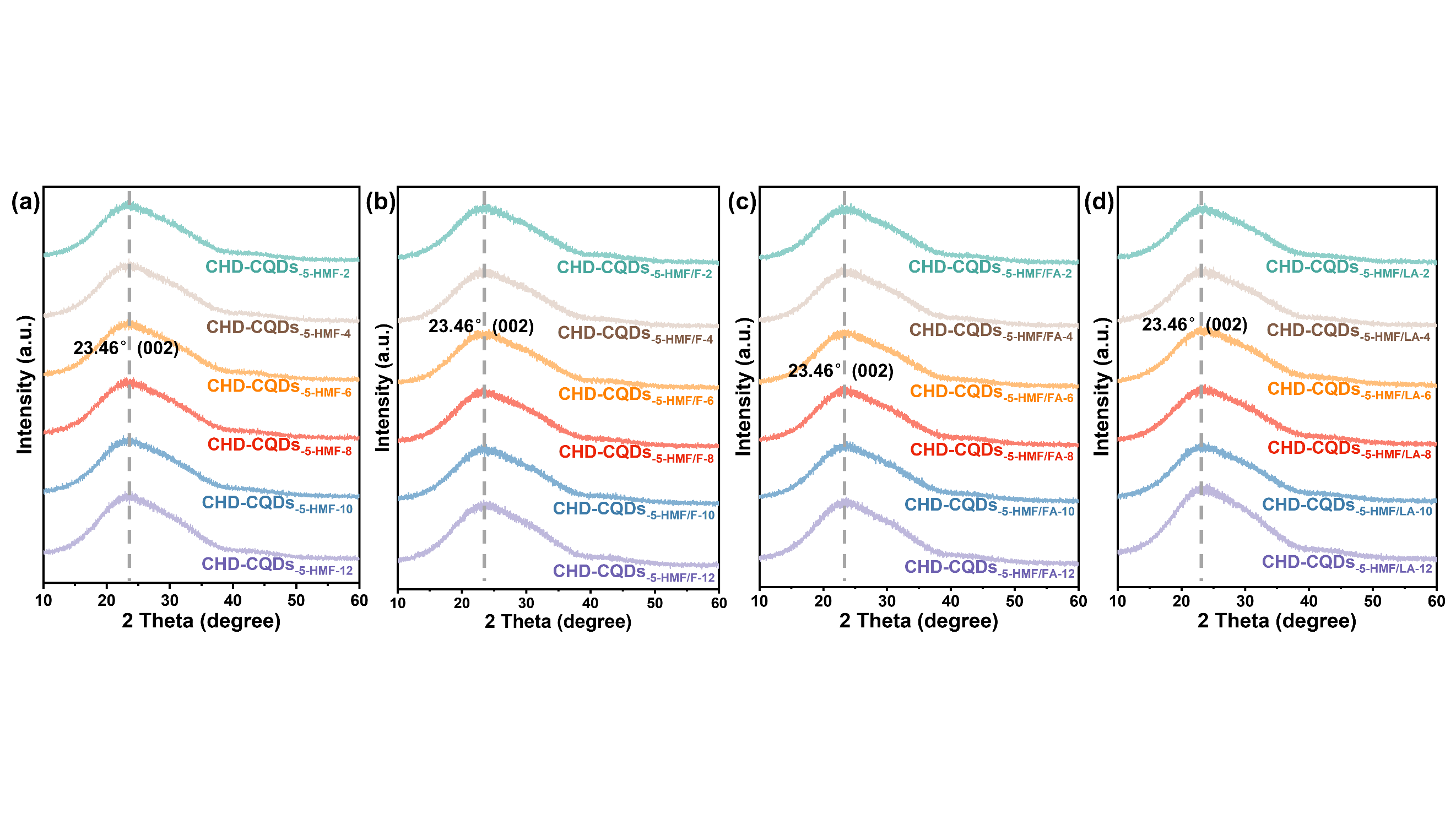


**Figure S8** XRD spectra of CHD-CQDs: (a) CHD-CQDs_-5-HMF_, (b) CHD-CQDs_-5-HMF/F_, (c) CHD-CQDs_-5-HMF/FA_, and (d) CHD-CQDs_-5-HMF/LA_


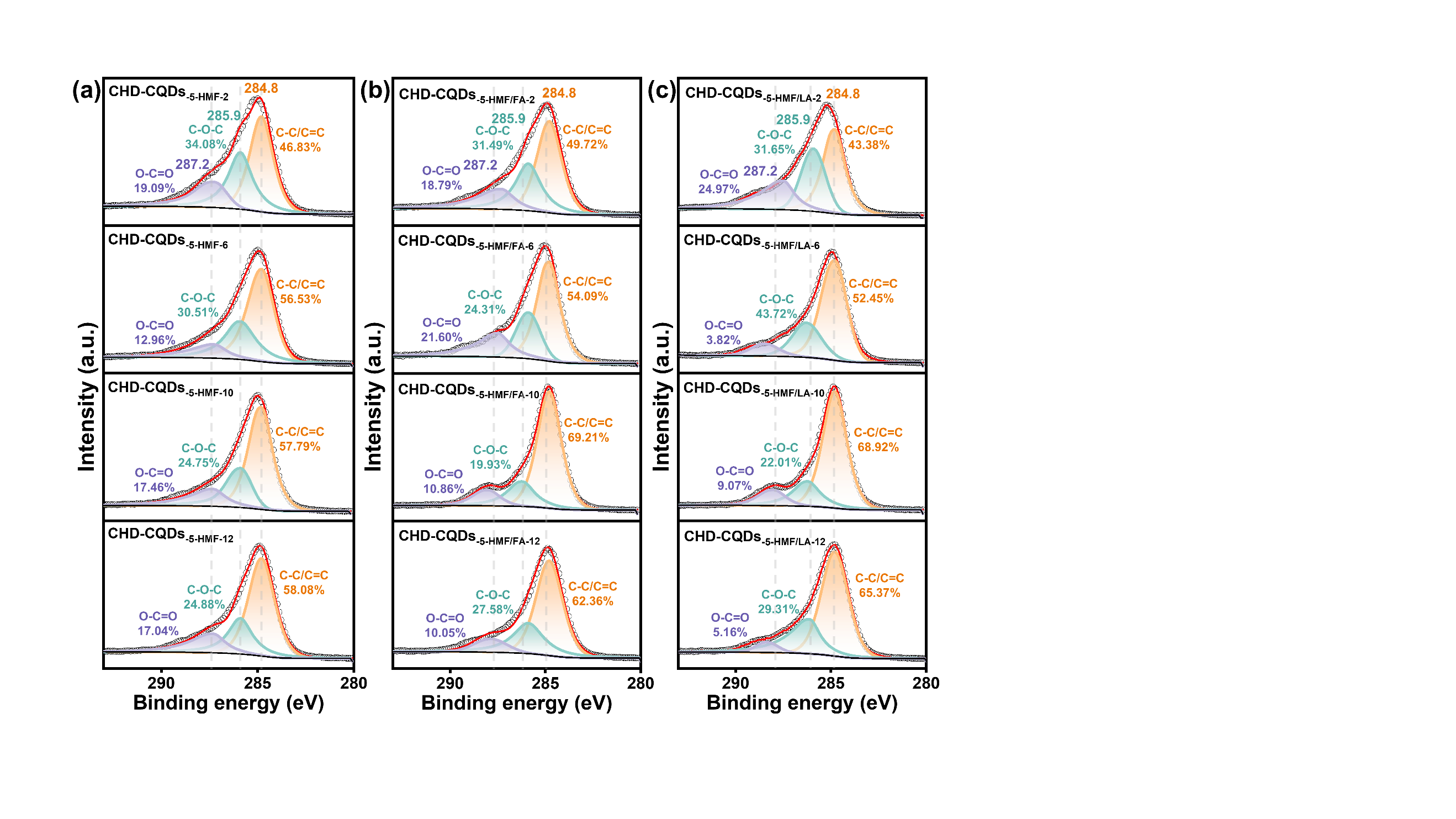


**Figure S9** XPS-C 1*s* fine spectra of CHD-CQDs: (a) CHD-CQDs_-5-HMF_, (b) CHD-CQDs_-5-HMF/FA_, (c) CHD-CQDs_-5-HMF/LA_


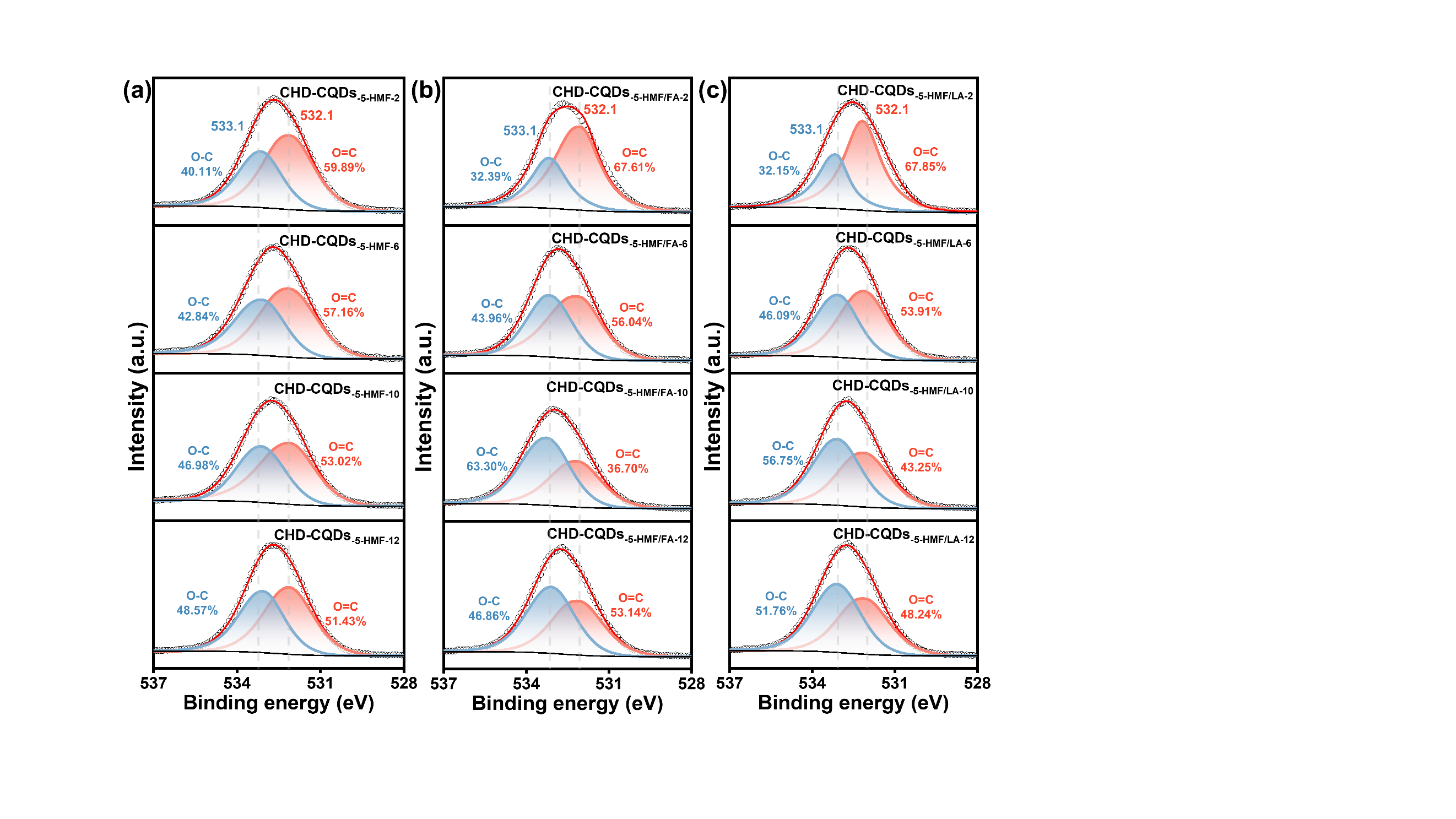


**Figure S10** XPS-O 1*s* fine spectra of CHD-CQDs: (a) (a) CHD-CQDs_-5-HMF_, (b) CHD-CQDs_-5-HMF/FA_, (c) CHD-CQDs_-5-HMF/LA_


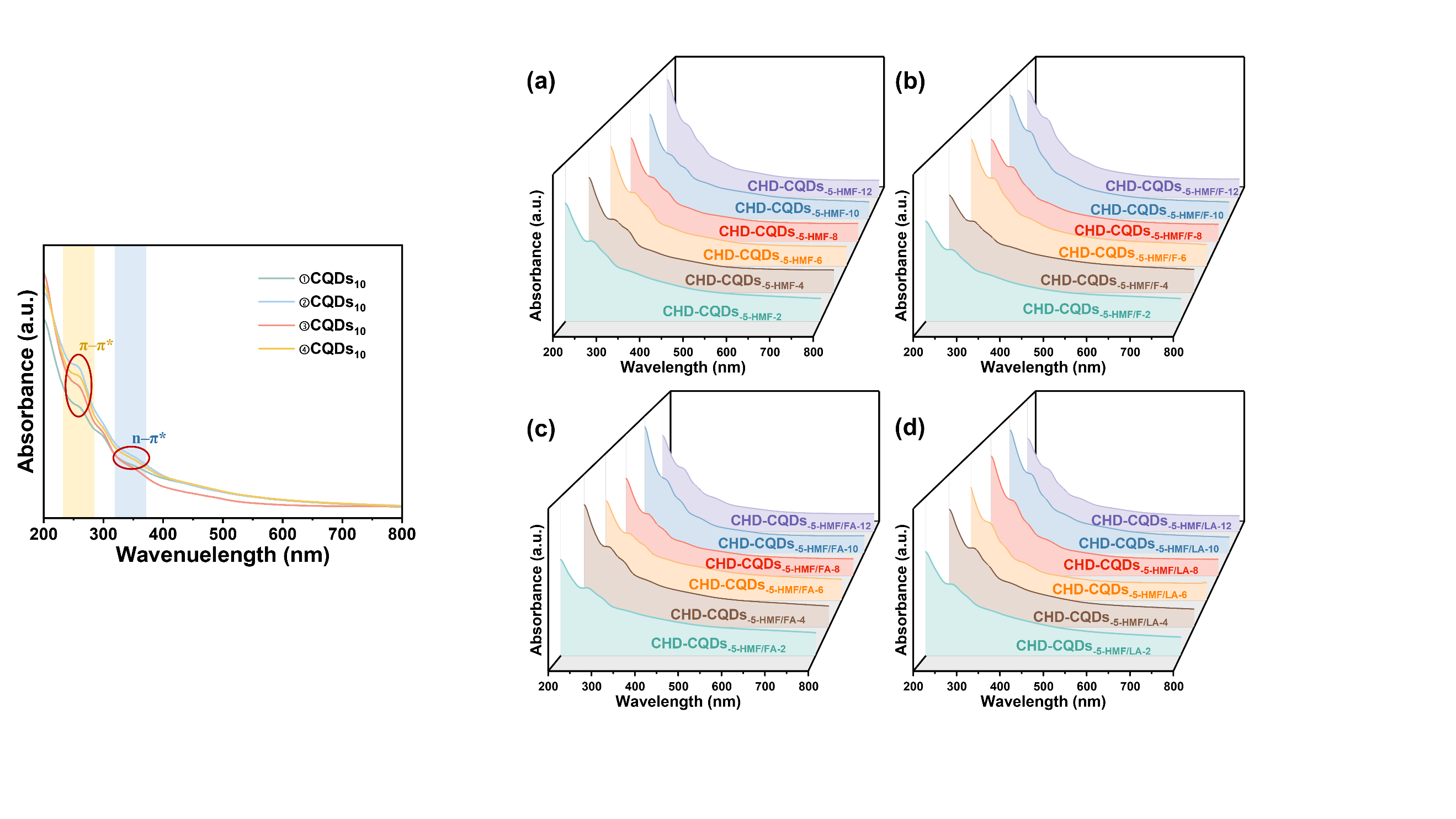


**Figure S11** UV-vis of CHD-CQDs: (a) CHD-CQDs_-5-HMF_, (b) CHD-CQDs_-5-HMF/F_, (c) CHD-CQDs_-5-HMF/FA_, and (d) CHD-CQDs_-5-HMF/LA_


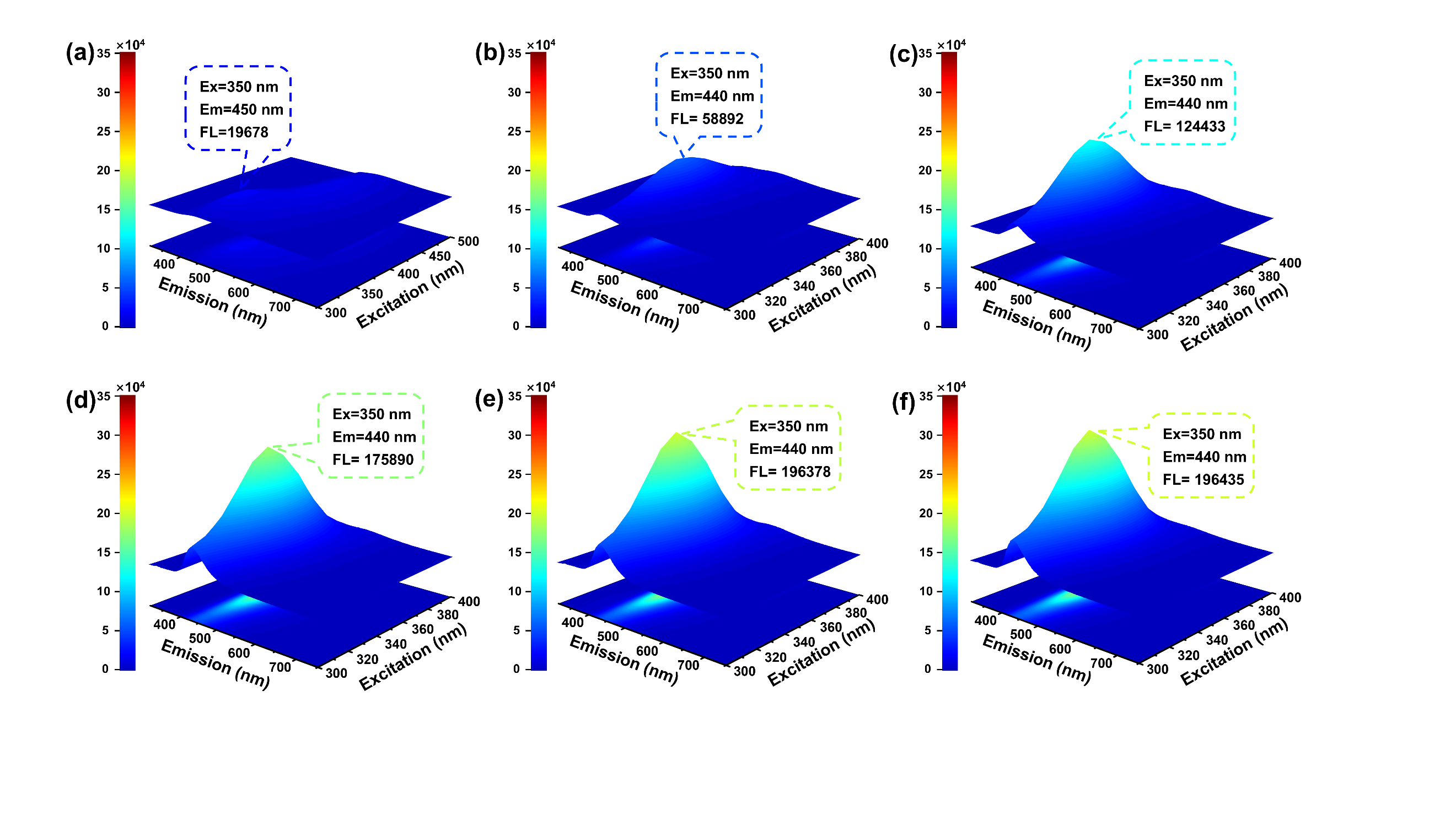


**Figure S12** Three-dimensional fluorescence spectra of CHD-CQDs-5-HMF: (a) CHD-CQDs_-5-HMF-2_, (b) CHD-CQDs_-5-HMF-4_, (c) CHD-CQDs_-5-HMF-6_, (d) CHD-CQDs_-5-HMF-8_, (e) CHD-CQDs_-5-HMF-10_, (f) CHD-CQDs_-5-HMF-12_


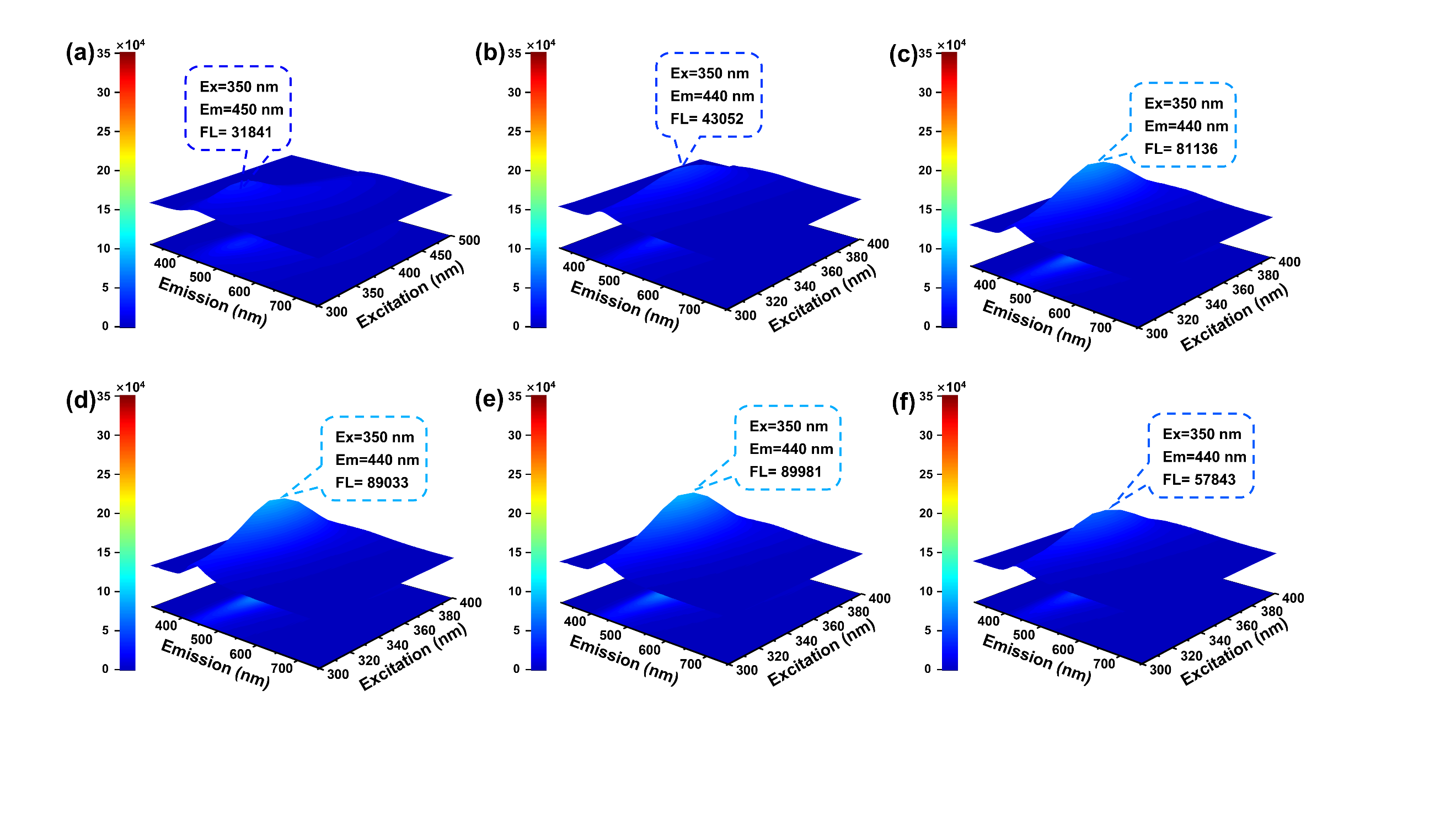


**Figure S13** Three-dimensional fluorescence spectra of CHD-CQDs_-5-HMF/F_: (a) CHD-CQDs_-5-HMF/F-2_, (b) CHD-CQDs_-5-HMF/F-4_, (c) CHD-CQDs_-5-HMF/F-6_, (d) CHD-CQDs_-5-HMF/F-8_, (e) CHD-CQDs_-5-HMF/F-10_, (f) CHD-CQDs_-5-HMF/F-12_


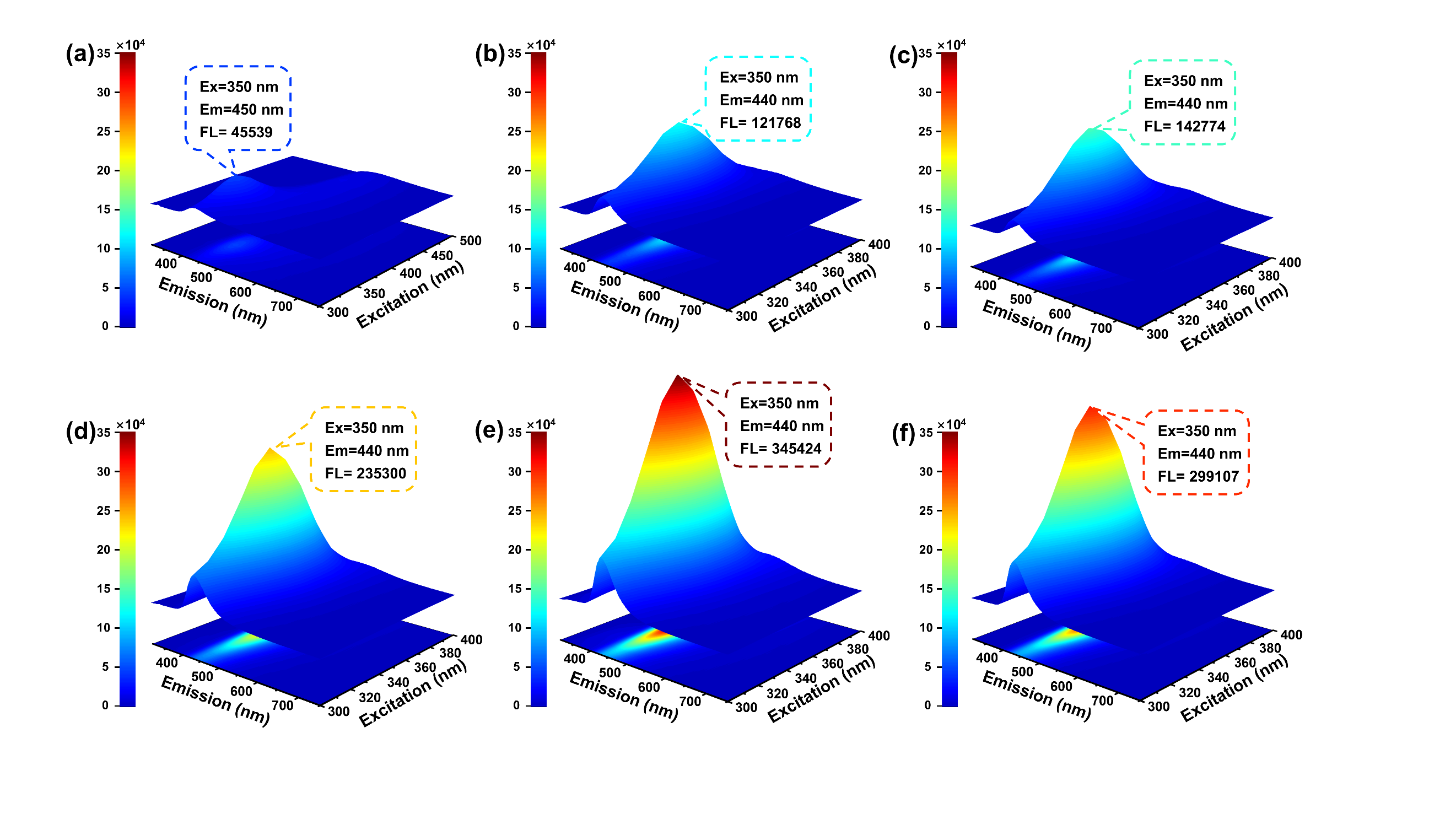


**Figure S14** Three-dimensional fluorescence spectra of CHD-CQDs_-5-HMF/FA_: (a) CHD-CQDs_-5-HMF/FA-2_, (b) CHD-CQDs_-5-HMF/FA-4_, (c) CHD-CQDs_-5-HMF/FA-6_, (d) CHD-CQDs_-5-HMF/FA-8_, (e) CHD-CQDs_-5-HMF/FA-10_, (f) CHD-CQDs_-5-HMF/FA-12_


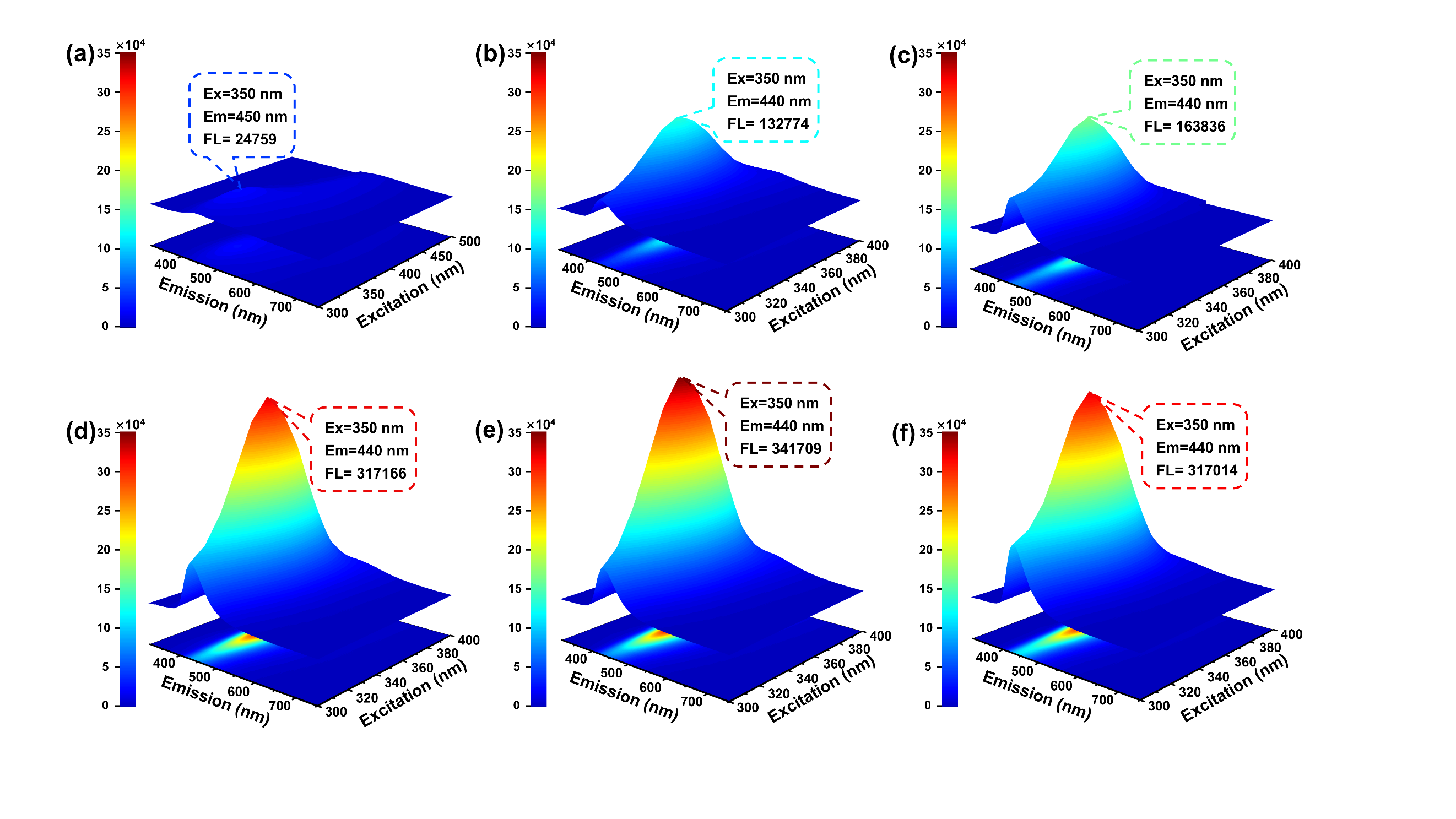


**Figure S15** Three-dimensional fluorescence spectra of CHD-CQDs_-5-HMF/LA_: (a) CHD-CQDs_-5-HMF/LA-2_, (b) CHD-CQDs_-5-HMF/LA-4_, (c) CHD-CQDs_-5-HMF/LA-6_, (d) CHD-CQDs_-5-HMF/LA-8_, (e) CHD-CQDs_-5-HMF/LA-10_, (f) CHD-CQDs_-5-HMF/LA-12_

**_
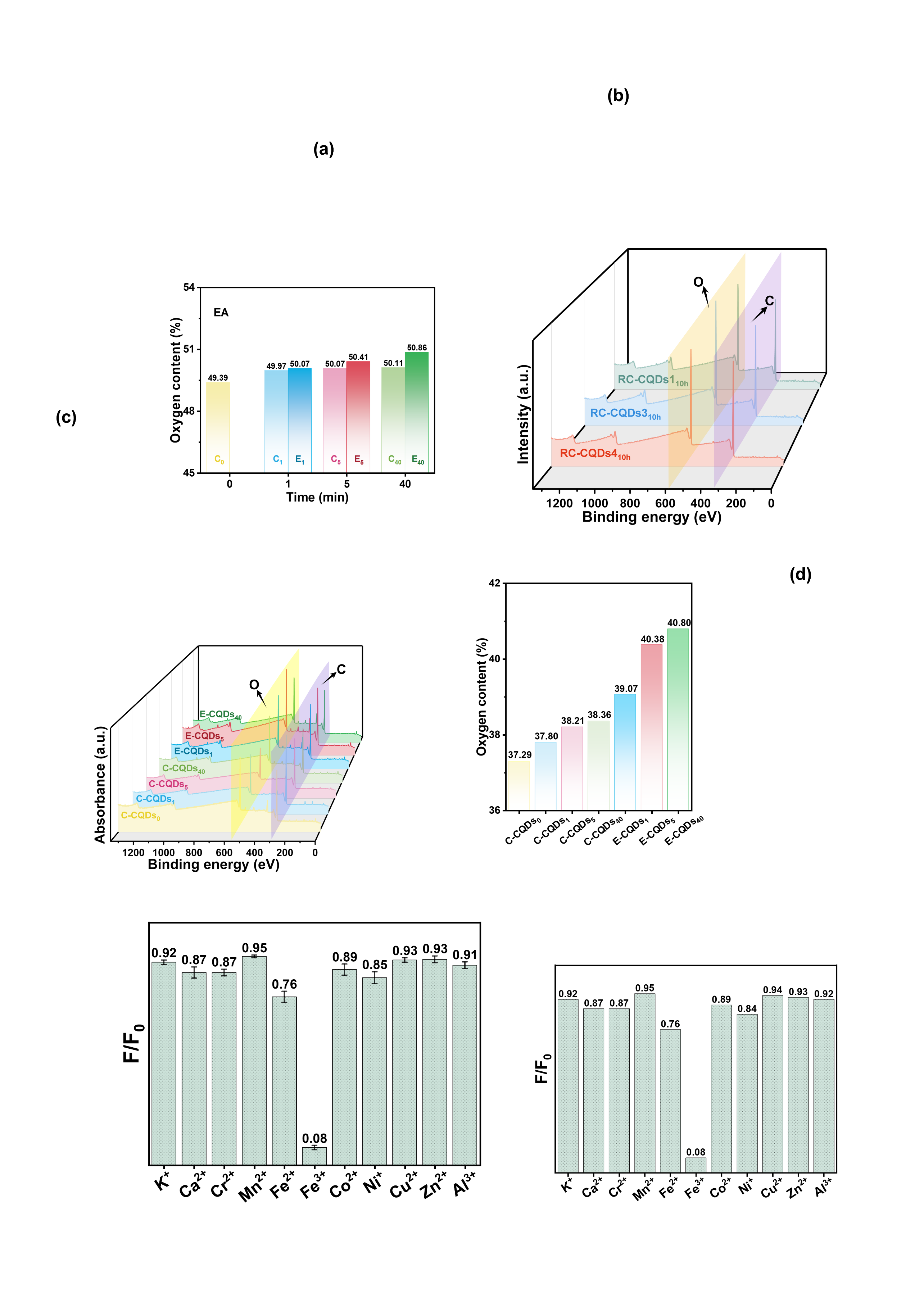
_**

**Figure S16** Effect of different metal ions on the fluorescence intensity ratio (F/F_0_) of CEM-CQDs_40_

[1] A. D. Becke, Density-Functional Thermochemistry. III. The Role of Exact Exchange. *J Chem Phys* **1993**, *98*, 5648.

[2] H. Zhao, J. H. Kwak, Z. Conrad Zhang, H. M. Brown, B. W. Arey, J. E. Holladay, Studying Cellulose Fiber Structure by SEM, XRD, NMR and Acid Hydrolysis. *Carbohydr Polym* **2007**, *68*, 235.

[3] K. S. Salem, N. K. Kasera, M. A. Rahman, H. Jameel, Y. Habibi, S. J. Eichhorn, A. D. French, L. Pal, L. A. Lucia, Comparison and Assessment of Methods for Cellulose Crystallinity Determination. *Chemical Society Reviews*, **2023**, *52*, 6417.

[4] D. R. da S. Souza, J. P. de Mesquita, R. M. Lago, L. D. Caminhas, F. V. Pereira, Cellulose Nanocrystals: A Versatile Precursor for the Preparation of Different Carbon Structures and Luminescent Carbon Dots. *Ind Crops Prod* **2016**, *93*, 121.

[5] C. Sarkar, A. R. Chowdhuri, A. Kumar, D. Laha, S. Garai, J. Chakraborty, S. K. Sahu, One Pot Synthesis of Carbon Dots Decorated Carboxymethyl Cellulose- Hydroxyapatite Nanocomposite for Drug Delivery, Tissue Engineering and Fe^3+^ Ion Sensing. *Carbohydr Polym* **2018**, *181*, 710.

[6] J. Woo, Y. Song, J. Ahn, H. Kim, Green One-Pot Preparation of Carbon Dots (CD)-Embedded Cellulose Transparent Film for Fe^3+^ Indicator Using Ionic Liquid. *Cellulose* **2020**, *27*, 4609.

[7] Z. Zhang, H. Chang, B. Xue, S. Zhang, X. Li, W. K. Wong, K. Li, X. Zhu, Near-Infrared and Visible Dual Emissive Transparent Nanopaper Based on Yb(III)–Carbon Quantum Dots Grafted Oxidized Nanofibrillated Cellulose for Anti-Counterfeiting Applications. *Cellulose* **2018**, *25*, 377.

[8] Z. Wang, F. Yuan, X. Li, Y. Li, H. Zhong, L. Fan, S. Yang, 53% Efficient Red Emissive Carbon Quantum Dots for High Color Rendering and Stable Warm White-Light-Emitting Diodes. *Advanced Materials* **2017**, *29*, 1702910.

[9] F. Yuan, Z. Wang, X. Li, Y. Li, ao Tan, L. Fan, S. F. Yang L Yuan, X. H. Li, Y. C. Li, L. Z. Fan, Z. B. Wang, Z. A. Tan, S. H. Yang, Near-Infrared and Visible Dual Emissive Transparent Nanopaper Based on Yb(III)–Carbon Quantum Dots Grafted Oxidized Nanofibrillated Cellulose for Anti-Counterfeiting Applications. *Advanced Materials* **2017**, *29*, 1604436.

[10] X. Li, X. Liu, Y. Su, T. Jiang, D. Li, X. Ma, Green Synthesis of Carbon Quantum Dots from Wasted Enzymatic Hydrolysis Lignin Catalyzed by Organic Acids for UV Shielding and Antioxidant Fluorescent Flexible Film. *Ind Crops Prod* **2022**, *188*, 115568.

[11] O. Losito, T. Netti, V. Kost, C. Annese, L. Catucci, T. Da Ros, V. De Leo, L. D’Accolti, Valorization of Soybean Peel-Derived Humins for Carbon Dot (CD) Production. *Materials* **2025**, *18*, 1865.

[12] S. Ding, Y. Gao, B. Ni, X. Yang, Green Synthesis of Biomass-Derived Carbon Quantum Dots as Fluorescent Probe for Fe^3+^ Detection. *Inorg Chem Commun* **2021**, *130*, 108636.

[13] Y. Qiu, D. Li, Y. Li, X. Ma, J. Li, Green Carbon Quantum Dots from Sustainable Lignocellulosic Biomass and Its Application in the Detection of Fe^3+^. *Cellulose* **2022**, *29*, 367.

[14] T. xiang Li, D. fang Zhao, L. Li, Y. Meng, Y. hui Xie, D. Feng, F. Wu, D. Xie, Y. Liu, Y. Mei, Unraveling Fluorescent Mechanism of Biomass-Sourced Carbon Dots Based on Three Major Components: Cellulose, Lignin, and Protein. *Bioresour Technol* **2024**, *394*, 130268.
